# Supplementary material for: Cancer Loyalty Card Study (CLOCS): feasibility outcomes for an observational case–control study focusing on the patient interval in ovarian cancer
Source: BMJ Open. 2023 Jun 13;13(6):e066022. doi: 10.1136/bmjopen-2022-066022 (PMC10277047; doi:10.1136/bmjopen-2022-066022)
Supplement: Supplementary data [file bmjopen-2022-066022supp001.pdf]

CLOCS
0000 0000 0000 0000
Cancer Loyalty Card Study

Participant number:
Attach barcode here

CRUK Cancer Loyalty Card Study (CLOCS)
Participant Questionnaire

Thank you for agreeing to complete this questionnaire.

- It should take no longer than 10-15 minutes to complete. You may, however, take as long as you wish.
- Please try to answer all of the questions, but if you do not wish to answer a question, you do not have to and you can skip to the next one.
- Your answers will be sent directly to the Imperial College research team and will be treated in strict confidence.
- There are four brief sections in this questionnaire:
    1. About you
    2. Your reproductive history
    3. Your medical history
    4. Your risk perception and health status
    5. About your loyalty card use
- Please tick the appropriate box where this option is given, or write in the box given.

e.g. ✓ or 1 3

Imperial College London

CANCER RESEARCH UK

Confidential once completed
Questionnaire V3, 05/05/2020

Brewer HR, *et al. BMJ Open* 2023; 13:e066022. doi: 10.1136/bmjopen-2022-066022

Participant number:
Attach barcode here

Today's Date: d d / m m / y y y y

1. About you

Date of birth: d d / m m / y y y y

Which of these best describes your ethnic group?

White
British ☐ Irish ☐ Any other white background ☐

Black or Black British
Caribbean ☐ African ☐ Any other Black background ☐

Asian or Asian British
Indian ☐ Pakistani ☐ Bangladeshi ☐ Any other Asian background ☐

Mixed
White and Black Caribbean ☐ White and Black African ☐ White and Asian ☐
Any other Mixed background ☐

Other Ethnic Groups
Chinese ☐ Any other ethnic group ☐ Prefer not to say ☐

What is your marital status?

Single / never married ☐ Married / living with partner ☐ Divorced / separated ☐
Widowed ☐ Prefer not to say ☐

What is your current height? ☐☐☐ cm or ☐ ft ☐☐ in

What is your current weight? ☐☐☐ kg or ☐☐ st ☐☐ lb

Including yourself, how many people live in your household? ☐ people

Brewer HR, *et al. BMJ Open* 2023; 13:e066022. doi: 10.1136/bmjopen-2022-066022

CLOCS
Cancer Loyalty Card Study

Participant number:
Attach barcode here

2. Your reproductive history

How old were you when you had your first period? ☐☐ years **or** I have never had a period ☐

Do you currently have regular periods?

Yes ☐ No ☐ **If no**, what was your age at menopause? ☐☐ years **or** irregular ☐

Not applicable, I have never had a period ☐

Have you ever been pregnant before? Yes ☐ No ☐

If yes:

How many pregnancies have you had that lasted less than 37 weeks? ☐☐

How many pregnancies have you had that lasted 37 weeks or more? ☐☐

How old were you when you first gave birth? ☐☐ years

How old were you when you last gave birth? ☐☐ years

Have you ever breastfed your children? Yes ☐ No ☐ Not applicable ☐

If yes:

For how many months (total for all children)? ☐☐ months

Have you had a hysterectomy (i.e. removal of your womb)? Yes ☐ No ☐

If yes:

At what age did you have a hysterectomy? ☐☐ years

Have you had tubal ligation (i.e. sterilization, tubes tied)? Yes ☐ No ☐

If yes:

At what age did you have tubal ligation? ☐☐ years

CLOCS
Cancer Loyalty Card Study

Participant number:
Attach barcode here

3. Your medical history

In the past 12 months, have you experienced any of these symptoms for an extended amount of time? (tick all that apply)

Feeling constantly bloated	☐	Loss of appetite	☐	Pain during sex	☐	Feeling full quickly	☐
Swollen Tummy	☐	Urge to pee more often or urgently than usual	☐	Constipation	☐	Feeling tired all the time	☐
Discomfort in lower tummy or pelvic area	☐	Persistent nausea	☐	Back pain	☐	Persistent indigestion	☐
Irregular periods	☐	Vaginal bleeding after menopause	☐	Unintentional weight loss	☐	None of these	☐

Did you use any over-the-counter (non-prescription) medication to manage these symptoms before seeing your GP?

Yes ☐ No ☐ Waited for the symptom to resolve on its own ☐ Not applicable ☐

How many times did you visit your GP in the past year? ☐☐ times

Have you ever been diagnosed with endometriosis? Yes ☐ No ☐ I don't know ☐

If yes:

At what age were you diagnosed with endometriosis? ☐☐ years

Have you ever taken aspirin regularly? Yes ☐ No ☐

If yes:

How often? Daily ☐ Weekly ☐ Less often ☐

For how long have you taken aspirin regularly? ☐☐ years and/or ☐☐ months

What dose of aspirin did you take?

Low dose (usually about 75mg/day) ☐ High dose (usually about 300mg/day) ☐ I don't know ☐

Have you ever used oral contraceptive pills (also called 'the pill' or 'birth control pill')? Yes ☐ No ☐

If yes:

For how long? ☐☐ years and/or ☐☐ months

Imperial College London

CANCER RESEARCH UK

Confidential once completed
Questionnaire V3, 05/05/2020

CLOCS
Cancer Loyalty Card Study

Participant number:
Attach barcode here

Your medical history continued

Have you ever used hormone replacement therapy? Yes ☐ No ☐

If yes:

For how long? ☐☐ years and/or ☐☐ months

Have you ever been diagnosed with cancer? Yes ☐ No ☐

If yes, what type? (tick all that apply):

Breast	☐	Melanoma	☐	Head and Neck	☐	Pancreas	☐	Uterus	☐
Bowel	☐	Non-Hodgkin Lymphoma	☐	Brain	☐	Leukemia	☐	Endometrial	☐
Lung	☐	Kidney	☐	Bladder	☐	Oesophagus	☐	Stomach	☐
Liver	☐	Myeloma	☐	Thyroid	☐	Ovarian	☐	Skin (non-melanoma)	☐

Other, please specify: ____

Please list any cancer(s) ticked above and the year in which you were diagnosed:

Type:________________________________ Year diagnosed: ☐☐☐☐

Type:________________________________ Year diagnosed: ☐☐☐☐

Do you have a mother, sister, or daughter with a history of <u>ovarian</u> cancer? Yes ☐ No ☐

If yes, in which relative(s)? Mother ☐ Sister ☐ Daughter ☐

Another sister or daughter, please specify relationship: ______________________ ☐ ______________________ ☐

Do you have a mother, sister, or daughter with a history of <u>breast</u> cancer? Yes ☐ No ☐

If yes, in which relative(s)? Mother ☐ Sister ☐ Daughter ☐

Another sister or daughter, please specify relationship: ______________________ ☐ ______________________ ☐

Imperial College London

CANCER RESEARCH UK

Brewer HR, *et al. BMJ Open* 2023; 13:e066022. doi: 10.1136/bmjopen-2022-066022

CLOCS
Cancer Loyalty Card Study

Participant number:
Attach barcode here

Your medical history continued

Have you ever regularly smoked cigarettes? Yes ☐ No ☐

If yes:

For how many years? ☐☐ years

Do you still smoke regularly? Yes ☐ No ☐

Have you ever regularly used vaping products? Yes ☐ No ☐

If yes:

For how many years? ☐☐ years

Do you still vape regularly? Yes ☐ No ☐

Due to the unprecedented Covid-19 (coronavirus) outbreak and lockdown started in March 2020 in the UK, our analysis of the past purchase information during the lockdown in the UK will be more complex than it would have been. We would like to be able to make sure we can control for the impact of Covid-19 on our analyses.

Have you had Covid-19 (coronavirus)?

Yes, diagnosed and recovered ☐ Yes, diagnosed and still ill ☐ Not formally diagnosed, but suspected ☐ No / Not that I know of ☐

If yes, please state the approximate date you presented with symptoms below:

d d / m m / y y y y

4. Your risk perception and health status

Compared to most other women your age, how likely do you think it is that you will get ovarian cancer at some time in your life?

Much lower than others ☐ Lower than others ☐ The same as others ☐ Higher than others ☐ Much higher than others ☐

How confident are you that you would notice an ovarian cancer symptom?

Not at all confident ☐ Not very confident ☐ Fairly confident ☐ Very confident ☐

Would you say your health is:

Excellent ☐ Very good ☐ Good ☐ Fair ☐ Poor ☐

Imperial College London

CANCER RESEARCH UK

Confidential once completed
Questionnaire V3, 05/05/2020

Brewer HR, *et al. BMJ Open* 2023; 13:e066022. doi: 10.1136/bmjopen-2022-066022

CLOCS
Cancer Loyalty Card Study

Participant number:
Attach barcode here

5. About your loyalty card use

At which food and pharmacy stores do you shop most regularly? (tick all that apply)

Tesco ☐ Sainsbury's ☐ Waitrose ☐ ASDA ☐ Boots ☐ Superdrug ☐

Co-op ☐ Morrison's ☐ Other, please specify: ____________________

At which food and pharmacy stores do you own a loyalty card? (tick all that apply)

Tesco ☐ Sainsbury's ☐ Waitrose ☐ ASDA ☐ Boots ☐ Superdrug ☐

Co-op ☐ Morrison's ☐ Other, please specify: ____________________

How often do you use loyalty cards when you go shopping?

Not at all ☐ Not very often ☐ Sometimes ☐ Often ☐ All the time ☐

How did you hear about this study?

From a patient ☐ Twitter ☐ Facebook ☐ Instagram ☐ Press ☐ Word of mouth ☐

Thank you for completing this form.

- Please return the survey in the freepost envelope (no stamp required).

Brewer HR, *et al. BMJ Open* 2023; 13:e066022. doi: 10.1136/bmjopen-2022-066022
