# Supplementary material for: Cancer Loyalty Card Study (CLOCS): feasibility outcomes for an observational case–control study focusing on the patient interval in ovarian cancer
Source: BMJ Open. 2023 Jun 13;13(6):e066022. doi: 10.1136/bmjopen-2022-066022 (PMC10277047; doi:10.1136/bmjopen-2022-066022)
Supplement: Supplementary data [file bmjopen-2022-066022supp002.pdf]

Cancer Loyalty Card Study (CLOCS): feasibility outcomes for an observational case-control study focusing on the patient interval in ovarian cancer

Hannah R. Brewer[1], Marc Chadeau-Hyam[2], Eric Johnson[2], Sudha Sundar[3], James M. Flanagan[1*] and Yasemin Hirst[4]

SUPPLEMENTARY MATERIAL

Supplementary Table 1: Ovarian Cancer Risk questionnaire item distribution and missing data

Risk factor questionnaire items	Cases		Controls	
	N	%	N	%
**Age (years)**	182	100.00	422	98.83
Missing	*0*	*0.00*	*5*	1.17
**Age at menarche (years)**				
08-11	38	20.88	95	22.25
12	43	23.63	89	20.84
13	39	21.43	101	23.65
14-19	57	31.32	138	32.32
Missing	*5*	2.75	*4*	0.94
**OC use**				
Never	43	23.63	50	11.71
Ever	138	75.82	377	88.29
Missing	*1*	0.55	*0*	0.00
**Duration of OC use (years)**				
0	52	28.57	56	13.11
1-5	59	32.42	140	32.79
>5	71	39.01	231	54.10
Missing	*0*	0.00	*0*	0.00
**Parity**				
Never	42	23.10	109	25.53
Ever	139	76.40	316	74.00
Missing	*1*	*0.50*	*2*	0.47
**No. full-term births**				

Brewer HR, *et al. BMJ Open* 2023; 13:e066022. doi: 10.1136/bmjopen-2022-066022

0	55	30.22	138	32.32
1	29	15.93	70	16.39
2	59	32.42	129	30.21
≥3	31	17.03	64	14.99
Missing	*8*	4.40	*26*	6.09
**No. non full-term births**				
0	125	68.68	244	57.14
1	29	15.93	97	22.72
2	10	5.49	29	6.79
>=3	6	3.30	26	6.09
Missing	*12*	6.59	*31*	7.26
**Age last birth**				
Never	49	26.92	128	29.98
<=25	25	13.74	45	10.54
26-30	44	24.18	83	19.44
>=31	57	31.32	153	35.83
Missing	*7*	3.85	*18*	4.22
**Breastfeeding**				
Never	94	51.65	202	47.31
Ever	87	47.80	220	51.52
Missing	*1*	0.55	*5*	1.17
**Breastfeeding duration (months)**				
Never	96	52.75	144	33.72
01-6	43	23.63	92	21.55
07-12	15	8.24	33	7.73
>12	27	14.84	79	18.50
Missing	*0*	0.00	*79*	18.50
**Tubal ligation**				
Never	154	84.62	376	88.06
Ever	22	12.09	51	11.94
Missing	*6*	3.30	*0*	0.00
**Endometriosis**				
Not diagnosed	170	93.41	393	92.04
Diagnosed	12	6.59	34	7.96
Missing	*0*	0.00	*0*	0.00
**Family history of breast cancer (first-degree relative)**				
None	150	82.42	366	85.71
Yes	31	17.03	60	14.05
Missing	*1*	0.55	*1*	0.23
**Family history of ovarian cancer (first-degree relative)**				
None	166	91.21	379	88.76
Yes	16	8.79	46	10.77

Brewer HR, *et al. BMJ Open* 2023; 13:e066022. doi: 10.1136/bmjopen-2022-066022

Missing	*0*	0.00	*2*	0.47
**BMI**				
Underweight (BMI < 18.5)	5	2.75	4	0.94
Normal (18.5 – 25)	75	41.21	138	32.32
Overweight (25-30)	50	27.47	131	30.68
Obese (>30)	44	24.18	125	29.27
Missing	*8*	4.40	*29*	6.79
**Aspirin use**				
Irregular or no use	159	87.36	390	91.33
Regular user of low-dose aspirin	18	9.89	28	6.56
Regular user of high-dose aspirin	3	1.65	1	0.23
Missing	*2*	1.10	*8*	1.87
**Menopausal status**				
Premenopausal	8	4.40	106	24.82
Postmenopausal	163	89.56	231	54.10
Missing	*11*	6.04	*90*	21.08
**Hysterectomy**				
Never	45	24.73	399	93.44
Ever	133	73.08	26	6.09
Missing	*4*	2.20	*2*	0.47
**HRT use**				
Never	123	67.58	343	80.33
Ever	58	31.87	84	19.67
Missing	*1*	0.55	*0*	0.00
**Duration of HRT use (years)**				
0	124	68.13	344	80.56
1-5	31	17.03	58	13.58
>5	27	14.84	21	4.92
Missing	*0*	0.00	*3*	0.70
**Smoking**				
Never	102	56.04	261	61.12
Ever	80	43.96	165	38.64
Missing	*0*	0.00	*1*	0.23
**Duration of Smoking (years)**				
0	102	56.04	261	61.12
1-10	27	14.84	55	12.88
>10-20	21	11.54	47	11.01
>20	32	17.58	60	14.05
Missing	*0*	0.00	*4*	0.94
**Vaping**				
Never	173	95.05	394	92.27
Ever	9	4.95	32	7.49
Missing	*0*	0.00	*1*	0.23

Brewer HR, *et al. BMJ Open* 2023; 13:e066022. doi: 10.1136/bmjopen-2022-066022

**Duration of Vaping (years)**				
0	173	95.05	394	92.27
1-5	7	3.85	27	6.32
>5	2	1.10	4	0.94
Missing	*0*	0.00	*2*	0.47
