# Supplementary material for: Cancer Loyalty Card Study (CLOCS): feasibility outcomes for an observational case–control study focusing on the patient interval in ovarian cancer
Source: BMJ Open. 2023 Jun 13;13(6):e066022. doi: 10.1136/bmjopen-2022-066022 (PMC10277047; doi:10.1136/bmjopen-2022-066022)
Supplement: Author's manuscript [file bmjopen-2022-066022.draft_revisions.pdf]

BMJ Open is committed to open peer review. As part of this commitment we make the peer review history of every article we publish publicly available.

When an article is published we post the peer reviewers' comments and the authors' responses online. We also post the versions of the paper that were used during peer review. These are the versions that the peer review comments apply to.

The versions of the paper that follow are the versions that were submitted during the peer review process. They are not the versions of record or the final published versions. They should not be cited or distributed as the published version of this manuscript.

BMJ Open is an open access journal and the full, final, typeset and author-corrected version of record of the manuscript is available on our site with no access controls, subscription charges or pay-per-view fees (http://bmjopen.bmj.com).

If you have any questions on BMJ Open's open peer review process please email
info.bmjopen@bmj.com

BMJ Open

Cancer Loyalty Card Study (CLOCS): feasibility outcomes for an observational case-control study focusing on the patient interval in ovarian cancer

Journal:	*BMJ Open*
Manuscript ID	bmjopen-2022-066022
Article Type:	Original research
Date Submitted by the Author:	27-Jun-2022
Complete List of Authors:	Brewer, Hannah; Imperial College London, Surgery and Cancer<br>Chadeau-Hyam, Marc; Imperial College London, MRC Centre for Environment and Health; Imperial College London, Epidemiology and Biostatistics, School of Public Health<br>Johnson, Eric; Imperial College London<br>Sundar, Sudha; University of Birmingham, Cancer Sciences<br>Flanagan, James; Imperial College London, Department of Surgery and Cancer<br>Hirst, Yasemin; University College London, Epidemiology and Public Health
Keywords:	Health informatics < BIOTECHNOLOGY & BIOINFORMATICS, Epidemiology < ONCOLOGY, Gynaecological oncology < ONCOLOGY

SCHOLARONE™
Manuscripts

I, the Submitting Author has the right to grant and does grant on behalf of all authors of the Work (as defined in the below author licence), an exclusive licence and/or a non-exclusive licence for contributions from authors who are: i) UK Crown employees; ii) where BMJ has agreed a CC-BY licence shall apply, and/or iii) in accordance with the terms applicable for US Federal Government officers or employees acting as part of their official duties; on a worldwide, perpetual, irrevocable, royalty-free basis to BMJ Publishing Group Ltd ("BMJ") its licensees and where the relevant Journal is co-owned by BMJ to the co-owners of the Journal, to publish the Work in this journal and any other BMJ products and to exploit all rights, as set out in our licence.

The Submitting Author accepts and understands that any supply made under these terms is made by BMJ to the Submitting Author unless you are acting as an employee on behalf of your employer or a postgraduate student of an affiliated institution which is paying any applicable article publishing charge ("APC") for Open Access articles. Where the Submitting Author wishes to make the Work available on an Open Access basis (and intends to pay the relevant APC), the terms of reuse of such Open Access shall be governed by a Creative Commons licence – details of these licences and which Creative Commons licence will apply to this Work are set out in our licence referred to above.

Other than as permitted in any relevant BMJ Author's Self Archiving Policies, I confirm this Work has not been accepted for publication elsewhere, is not being considered for publication elsewhere and does not duplicate material already published. I confirm all authors consent to publication of this Work and authorise the granting of this licence.

For peer review only

Cancer Loyalty Card Study (CLOCS): feasibility outcomes for an observational case-control study focusing on the patient interval in ovarian cancer

Hannah R. Brewer[1], Marc Chadeau-Hyam[2], Eric Johnson[2], Sudha Sundar[3], James M. Flanagan[1*] and Yasemin Hirst[4]

1. Division of Cancer, Department of Surgery and Cancer, Imperial College London, London

2. School of Public Health, Imperial College London, London

3. Institute of Cancer and Genomic Sciences, University of Birmingham, Birmingham

4. Department of Behavioural Science and Health, University College London, London

***Address for correspondence:**

Dr. James M. Flanagan

Division of Cancer, Department of Surgery and Cancer,

Faculty of Medicine, Imperial College London

4th Floor IRDB, Hammersmith Campus, Du Cane Road, London W12 0NN

Tel: 020 75942127; email: j.flanagan@imperial.ac.uk;

Word Count

Body: 4141

Abstract: 289

For peer review only - http://bmjopen.bmj.com/site/about/guidelines.xhtml

ABSTRACT

Objectives: Ovarian cancer symptoms are often non-specific and can be normalised before patients seek medical help. The Cancer Loyalty Card Study (CLOCS) investigates self-management behaviours of ovarian cancer patients prior to their diagnosis using loyalty card data collected by two UK-based high street retailers. Here, we discuss the feasibility outcomes for this novel research.

Design: Observational case-control study.

Setting: Control participants were invited to the study using social media and other sources from the general public. Once consented, control participants were required to submit proof of identification (ID) for their loyalty card data to be shared. Cases were identified using unique NHS numbers (a proxy for ID) and were recruited through 12 NHS tertiary care clinics.

Participants: Women in the UK, 18 years or older, with at least one of the participating high street retailers' loyalty cards. Those with an ovarian cancer diagnosis within two years of recruitment were considered cases, and those without an ovarian cancer diagnosis were considered controls.

Primary Outcome Measures: Recruitment rates, demographics of participants and identification of any barriers to recruitment.

Results: In total, 182 cases and 427 controls were recruited with significant differences by age, number of people in participants' households and the geographical region in the UK. However, only 37% (n=160/427) of control participants provided sufficient ID details and 81% (130/160) matched retailers' records. The majority of the participants provided complete responses to the 24-Item Ovarian Risk Questionnaire.

Conclusions: Our findings show that recruitment to a study aiming to understand self-care behaviours using loyalty card data is challenging but feasible. The general public were willing to share their data for health research. Barriers in data sharing mechanisms need to be addressed to maximise participant retention.

Trial registration: ISRCTN 14897082, CPMS 43323, NCT03994653.

For peer review only - http://bmjopen.bmj.com/site/about/guidelines.xhtml

STRENGTHS AND LIMITATIONS OF THIS STUDY

Main Strengths:

- This study is the first of its kind to investigate transactional data for cancer outcomes;
- it has established data governance structures and methods to comply with GDPR and privacy policies for using retailer transactional data;
- and it provides a pre-existing prospectively collected data set free from recall biases which is a common bias in case-control studies.

Main Limitations:

- Recruitment happened during the COVID-19 pandemic which affected recruitment rates, particularly for cases who were recruited through NHS clinics;
- Increasing age was negatively associated with participation rates, particularly in the control population, requiring targeted recruitment to older participants.

KEYWORDS:

Observational case control study; feasibility ; recruitment ; transactional data ; cancer ; diagnosis

For peer review only - http://bmjopen.bmj.com/site/about/guidelines.xhtml

INTRODUCTION

Approximately 7500 new cases of ovarian cancer are diagnosed in the UK each year, and it is estimated that ovarian cancer incidence rates will rise by 15% in the UK between 2014 and 2035 [1]. Unfortunately, 23% of ovarian cancer patients in 2018 were diagnosed at Stage IV [2] when treatment options are less than optimal for survival. Late-stage ovarian cancer diagnosis is often associated with delayed patient presentation and a longer diagnostic interval due to non-specific cancer symptoms (e.g., feeling bloated, indigestion, feeling full, abdominal pain). While there has been progress in efforts to reduce diagnostic delays using routine blood tests (i.e., CA125) [3] there is little evidence focusing on women's self-care behaviours associated with managing non-specific symptoms.

The Cancer Loyalty Card Study (CLOCS) investigates the period between the presentation of the first symptom(s)/sign(s) leading up to the first presentation at health care providers (i.e., the patient interval) of ovarian cancer patients to better understand self-care behaviours using past purchase information collected through loyalty cards at two high street retailers in the UK [4]. This project aims to better understand whether self-care behaviours can be detected at an early stage and facilitate earlier presentations to healthcare providers. We have previously reported in a proof-of-concept study that loyalty card data may be used to identify an increase in painkiller and indigestion medication purchases among ovarian cancer patients [5]. The proof-of-concept study warranted testing this hypothesis in a large-scale case-control study design aiming to include 500 ovarian cancer patients (cases) and 500 individuals without an ovarian cancer diagnosis (controls) [4].

Flanagan et al demonstrated the key information governance and ethical considerations of recruiting participants to a project that is requesting consent for transactional data to be used in health research [5]. These included separate consent forms for the study and data

For peer review only - http://bmjopen.bmj.com/site/about/guidelines.xhtml

requests for each retailer, clear communication with potential participants about which information is being collected, stored, how it is being used, how long, and data breach risks, if any. Loyalty card ownership was also identified as a logistic barrier. Thus, ownership was recommended as a prerequisite of participation in the study to avoid data loss or invalidated consents. In terms of data reliability among cases, it was also recommended that patients should be recruited to the study close to their initial diagnosis for researchers to have a retrospective purchase history capturing self-care behaviours before ovarian cancer diagnosis.

Furthermore, recruitment and participant retention to case-control studies are not without challenges; they have often been criticised for potential selection bias and representativeness of the control participants against the case participants [6]. Traditional recruitment strategies often involve recruitment through healthcare organisations, random digit dialling and postal invitations, recruitment via family and friends of patients [7]. In CLOCS, due to low prevalence of ovarian cancer in the UK, a multi-site recruitment approach for cases in the UK was proposed with social media recruitment of controls to minimise selection bias and maximise representativeness [4]. However, while the patients' willingness to take part may be higher for cancer research, it was also deemed necessary to improve public acceptability among potential control participants by raising awareness and improving understanding using an accessible communication format, i.e., a tailored website (www.clocsproject.org.uk).

The aim of this paper is to present the feasibility outcomes of CLOCS which aimed to recruit 1000 women with and without ovarian cancer in the UK, describe feasibility of a large-scale case-control study by assessing participant rates, characteristics and any possible changes to the study design that could optimise data collection in the future. In particular, we discuss

For peer review only - http://bmjopen.bmj.com/site/about/guidelines.xhtml

the barriers and facilitators of recruiting control participants to a case-control study through social media platforms and case participants using ovarian cancer patient clinics in NHS trusts in light of the COVID-19 pandemic.

METHODS

Design

The aims and design of CLOCS have previously been reported in the study protocol [4]. CLOCS is an observational case-control study where participants are women in the UK, aged 18 years or older, who hold a loyalty card at either or both of the participating high street retailers (HSR1 and HSR2). Participants with a diagnosis of ovarian cancer were considered cases, and those without a previous ovarian cancer diagnosis were considered controls.

Setting/s

Cases were recruited from 12 NHS clinic sites in England, Wales, and Scotland. Eligible patients were identified by each site's recruitment team and presented with information about the study in paper format. If they decided to take part, they were given the consent form, a risk factor questionnaire, and a clinical questionnaire, which was completed by their clinical care team, to either complete in the clinic or take with them to complete at another time. Completed forms were then posted to the CLOCS main research site.

In response to the COVID-19 pandemic, an amendment was submitted and approved by the NHS Research Ethics Committee (20 September 2020, 19/NW.0427-SA1) to allow clinics to

For peer review only - http://bmjopen.bmj.com/site/about/guidelines.xhtml

recruit eligible ovarian cancer patients over the phone, post them the study documents to complete where they were shielding, and post completed forms to the main research site.

Controls were recruited primarily using paid/non-paid web-based methods, snowball sampling, a pilot targeted email campaign from one of the participating high street retailers to its users, and word of mouth. These methods included paid Facebook advertising, distribution of study blogs on the study website, study twitter (@CLOCS_Imperial), VOICE Imperial College London[8], and widely distributed webinars that aimed to address barriers in CLOCS participation. In October 2021, a pilot email was sent to 1053 cardholders from one of the retailers to test the impact of sending invitations for CLOCS and measure the response rate to invitations.

Procedures

Consent + Questionnaire Completion (Step 1)

Each potential participant was provided with a detailed information sheet and privacy policy to facilitate informed decision making. This was followed by study consent, the study questionnaire, and a clinical questionnaire for cases. The CLOCS participant consent form consisted of two sections. In the first section, participants consented to take part in the study which provided information on data retention, how data is being handled for the study, how long, and how to withdraw. In the second section, participants provided consent for the research team to request their purchase history from the participating retailer(s) using the exact numbers and names on their loyalty cards. Contact details were collected to clarify loyalty card details in the event their details did not match the retailers' records.

For peer review only - http://bmjopen.bmj.com/site/about/guidelines.xhtml

Participants were given the option to consent to be re-contacted to take part in future related studies.

Case participants completed paper-based forms which were then posted back to the research team for secure data entry. Control participants were asked to complete a web-based secure study form including the information sheet, the consent form and risk factor questionnaire on the study's website (www.clocsproject.org.uk)

Substantial Amendment - ID verification (Step 2)

In September 2020, the CLOCS research team received approval from the North West - Greater Manchester South Research Ethics Committee (dated 20 September 2020, 19/NW/0427-SA1) to request identity verification details including a visual proof of ID (e.g., passport), and a proof of address (e.g., utility bill) from participants. This was a development during the research recruitment process before data sharing agreements with HSR1 and HSR2 were established. Under Article 15 in the General Data Protection Regulation (GDPR) in the UK, individuals have the right to access and request a copy of their data, often referred to as a subject access request [9]. There is growing literature on impersonating to take part in research for financial gains and/or trying to get access to sensitive information [10]. Thus, the aim of the ID verification step was to ensure that those participating in the study were not completing the forms on behalf of someone else and could be verified against the high street retailer's records. Therefore, control participants who took part in the study online were asked to provide a copy of a photo ID and proof of address, using a unique and secure weblink, after Step 1 was completed. If they did not complete Step 2 at the time of the participation, they were sent a maximum of three reminder emails, which

For peer review only - http://bmjopen.bmj.com/site/about/guidelines.xhtml

included their unique upload link. All ID verification documents were encrypted using private and public keys and kept until data requests from the retailers were completed or when the study was complete, whichever came first. Control participants who took part prior to September 2020 were sent unique secure links to consider uploading this information so that the research team could request their purchase history data from the retailers. If they did not upload the documents after their reminders, no requests were made from the retailers on their behalf. Identification of cases in NHS clinics using unique NHS numbers was sufficient for ID verification. Details of the transfer of participant purchase history between the research team and retailers are described in the protocol [4].

Recruitment Status

Recruitment was opened for ovarian patients from 1st November 2019 and for control participants from 1st July 2020. Recruitment was suspended from 31st Jan 2022 (the original planned closing date) pending funding decisions, and was finally closed on 28th July 2022.

Patient and Public Involvement (PPI)

PPI informed the design and the recruitment strategies throughout CLOCS. We presented two public facing seminars in December 2020 aiming to engage with the wider audience about CLOCS and changes in the participation criteria using ID verification and in March 2021 during Ovarian Cancer Awareness month aiming to discuss the potential impact for ovarian cancer patients. Furthermore, we held three scientific meetings annually inviting academics, patient representatives, and participating high street retailers.

Feasibility outcomes measures

For peer review only - http://bmjopen.bmj.com/site/about/guidelines.xhtml

The following measures were collected and used to assess feasibility outcomes of CLOCS based on guidance on reporting feasibility outcomes and the reporting of observational studies in Epidemiology (STROBE).

Participant Descriptive Characteristics: Date of birth was collected and re-categorised in 10-year age groups for reporting. Other characteristics included ethnicity, the number of people in the participant's household (1 to ≥5 persons), self-reported loyalty card usage (not at all/not very often/sometimes/often/all the time/missing), loyalty card membership for the retailers (HSR1, HSR2, both and neither) included in the study, and family history of ovarian and/or breast cancer (Yes/No/Missing). We included 17 different ethnic backgrounds to be recorded, due to the small number participants in sub-populations, we categorise this as white/non-white/prefer not to say and missing.

The questionnaire was amended to include a question about whether participants had been diagnosed with COVID-19 (responses limited to the following: diagnosed and recovered, diagnosed and still ill, suspected but not formally diagnosed/did not have COVID-19) at the time of the study participation.

Further data included the Index of Multiple Deprivation (IMD) quintiles based on the postcodes of the stores where purchases were made ranging from most deprived (1) to least deprived (5). The location where the purchases were made was included based on the nine UK Regions (Table 3), excluding any purchases that were made in Northern Ireland due to small numbers (n<5).

Ovarian Cancer Risk Questionnaire (24 Items)

For peer review only - http://bmjopen.bmj.com/site/about/guidelines.xhtml

Participants completed a short questionnaire about ovarian cancer risk factors including ethnicity, marital status, body mass index (BMI), age at menarche, menopausal status, age at menopause, parity, breastfeeding, hysterectomy, tubal ligation, cancer history, endometriosis, aspirin use, oral contraceptive (OC) use, hormone replacement therapy (HRT) use, family history of ovarian and breast cancers, vaping, and cigarette smoking. The questionnaire requested information about the symptoms experienced (if any) and number of visits to the general practitioner in the year leading up to participation for controls and cancer referral or diagnosis for cases. All responses were voluntary.

Participant Retention

Participant retention was measured at three stages. The first stage was the number of participants with a valid consent and completed ovarian cancer risk questionnaire. Participant retention was calculated based on withdrawals and valid consent (e.g. provided consent for retailers).

The second stage assessed the number of control participants with valid identity verification details. Identity verification was recorded as ID verified or not ID.

The last stage of retention was assessed based on the confirmation of the records with the retailers following transactional data requests. If participants' reported card details were the same as the retailers' and data was received, this was recorded as complete participation. If their reported card details did not match the retailers' records, we recorded these participants as unmatched.

Statistical Analysis

Participation and data retention was recorded in a CONSORT diagram. A chi-square test was used to assess the differences between cases and controls based on the following

For peer review only - http://bmjopen.bmj.com/site/about/guidelines.xhtml

characteristics: age (10 year age-groups), ethnicity, number of people in the household, loyalty card ownership, and the location and IMD quintile of the stores where transactions took place. Each postcode for the stores where individuals purchased items were converted to the UK regions and IMD deciles using the English Indices of Deprivation 2019 [11]. These were then recoded into quintiles ranging from most deprived to least deprived [12]. If the sample size was less than 5, groups were merged together to ensure safe data reporting. Missing data and questionnaire completion rates were calculated.

RESULTS

Recruitment Timeline

The cumulative recruitment of CLOCS cases and controls is shown in Figure 1. CLOCS recruitment began in December 2019, and at the beginning of the COVID-19 pandemic in the UK in March 2020, recruitment was paused to investigate the impact of the pandemic on recruitment while patients were not able to attend clinics.

Active control recruitment was also paused, however, opportunistic recruitment occurred through secondary sources. The changes were implemented, and patient recruitment resumed from September 2020. Facebook Advertising was used to recruit control participants from 17th September 2020 - 26th April 2021.

Participant Characteristics (included in ovarian cancer risk analysis)

The characteristics of CLOCS participants included in the final dataset for ovarian cancer risk analysis are shown in Table 1.

For peer review only - http://bmjopen.bmj.com/site/about/guidelines.xhtml

For peer review only

Table 1: Participant Characteristics

Characteristic	Cases (N= 182)		Controls (N=427)		
	N	%	N	%	*P*
**Age**					
18-39	6	3.6	61	16.2	<0.001
40-49	7	4.7	96	23.8	
50-59	32	21.3	125	31.8	
60-69	64	36.7	107	21.2	
≥70	73	33.7	33	5.9	
missing	0	0.0	5	1.2	
**Ethnicity**					
White	173	95.1	400	93.7	0.392
Non-white	8	4.4	11	2.6	
Prefer not to say	0	0.0	0	0.0	
Missing	1	0.5	16	3.7	
**Number of people in the household**					
1	35	22.2	45	14.5	<0.001
2	78	52.1	120	40.5	
3	17	11.8	62	20.9	
4	10	6.9	45	15.2	
≥5	2	1.4	24	8.1	
missing	8	5.6	2	0.7	
**Family history of breast or ovarian cancer**					
No	139	76.4	327	76.6	1.00
Yes	43	23.6	99	23.2	
Missing	0	0.0	1	0.2	
**Loyalty Card**					
HSR1 only	48	26.4	169	39.6	0.001
HSR2 only	44	24.2	116	27.2	
Both HSR1 and HSR2	86	47.3	142	33.3	
Neither	4	2.2	0	0.0	
**Loyalty Card Use**					
Not at all	1	0.5	2	0.5	0.449
Not very often	9	4.9	10	2.3	
Sometimes	18	9.9	42	9.8	
Often	42	23.1	117	27.4	
All the time	109	59.9	256	60.0	
Missing	3	1.6	0	0.0	

For peer review only - http://bmjopen.bmj.com/site/about/guidelines.xhtml

Cases

NHS recruitment sites reported that 306 patients were eligible and interested to take part in CLOCS. Of those, 183 patients (59%) returned their completed consent form and questionnaires. After exclusion, 182 were included in the final dataset for ovarian cancer risk analysis. About 70 % of cases who took part in CLOCS were diagnosed from January 2020 onward (Figure 2).

Most cases were 60 years or older (70.4%). The majority of the cases identified themselves as white ethnic background (95.1%), and the rest of the cases were grouped together as non-white ethnic background due to small numbers (4.4%). Over half of case participants reported living in a two-member household (52.1%), followed by 22.2% in a single-member household, and 20.1% living in a household of three or more people. About 25% of cases had a family history of ovarian and/or breast cancer in first-degree, female relative.

Controls

In total, there were 657 online submissions of the secure online questionnaire (See Figure 2). Of those, 441 (61.7%) were considered valid control participants once duplicates and test submissions were removed. There were 14 participants who were excluded from the study; 10 withdrew from the study, and 4 were ineligible. Therefore, 427 control participants were included in the final dataset for ovarian cancer risk analysis.

Compared to case participants, controls were younger with a larger proportion in age groups 40-49 years (23.8%) and 50-59 years (31.8%). There were also significantly fewer

For peer review only - http://bmjopen.bmj.com/site/about/guidelines.xhtml

For peer review only

control participants recruited in the older age groups 60-69 years (21.2%) and 70 years and older (5.9%) compared with case participants (p<0.05). Similar to cases, most controls reported living in a two-member household (40.5%), 14.5% in a single-member household, and 44.2% in a household with three or more individuals. Also, 76.6% of control participants reported having a first-degree, female relative with ovarian and/or breast cancer.

Participant Retention Rates

The recruitment pathways for cases and controls and how participants were retained within the study are reported in the CONSORT diagram in Figure 3.

While case participants did not require additional identity verification, there were four cases who did not provide loyalty card details, and therefore, 178 cases were eligible for data requests from the retailers. In total 134 (73.6%) case participants were eligible for data requests from the HSR1 and 130 (71.4%) case participants from HSR2. In the end, transactional history data were received for 158 cases (88% case participant data retention rate). (Table 2)

Table 2 - Number of people included in the transactional data analysis by age and retailers

	HSR1				HSR2			
**Age**	**Cases (N=134)**		**Eligible Controls (N=120)**		**Cases (N=130)**		**Eligible Controls (N=107)**	
	**Matched**	**%**	**Matched**	**%**	**Matched**	**%**	**Matched**	**%**
**18-49**	9	6.7	41	34.2	10	7.6	28	26.2
**50-59**	19	14.2	31	25.8	26	20.0	25	23.4
**60-69**	43	32.1	25	20.8	43	33.1	20	18.7
**70+**	58	43.3	9	7.5	48	36.9	10	9.3
**Total**	129	96.3	106	88.3	127	97.7	83	77.6

Eligible = ID verified

For peer review only - http://bmjopen.bmj.com/site/about/guidelines.xhtml

To maximise the number of ID verified control participants, over 700 email reminders were sent to participants asking them to complete the ID verification step. As of 31 January 2022, 160 (37.5%) control participants provided sufficient ID verification and were eligible for purchase history requests on their behalf. At the end of the study, 137 control participant (85.6% control participant data retention rate) card details matched retailer records, allowing the research team to receive up to six years of their past transactional data (See Table 3). Only 51% of control participants had records for both retailers. The association between age distribution and ID verification among control participants was not significant (p=0.80).

Representativeness based on the store postcodes recorded on loyalty cards

Based on the transactions recorded on loyalty cards, we identified 697 transactions for cases and 1127 transactions for controls in nine UK Regions, excluding Northern Ireland. The proportion of the distribution of the purchases carried out by cases and controls in each UK region were significantly different, $X^2(8, 1824) = 177.22$, $p<0.001$ (See Table 3). More than five percentage points difference between cases and controls were recorded in transactions at East of England and Yorkshire and Humber (6.9% vs 18.5%), Midlands (4.3% vs 12.1%), South East (27.7% vs. 17.6%), and Wales (15.1% vs 3.8%) respectively. The largest proportion of purchases for both cases and controls were collected in South East.

Table 3. The number of transactions recorded on loyalty cards among participants in each UK region.

For peer review only - http://bmjopen.bmj.com/site/about/guidelines.xhtml

UK Regions[t]	Transactions			
	Cases		Controls	
	N *	%	N*	%
**East of England & Yorkshire and Humber**	48	6.9	209	18.5
**London**	104	14.9	192	17.0
**North East**	24	3.4	36	3.2
**North West**	47	6.7	87	7.7
**Midlands**	30	4.3	136	12.1
**Scotland**	85	12.2	82	7.3
**South East**	193	27.7	198	17.6
**South West**	61	8.8	144	12.8
**Wales**	105	15.1	43	3.8
**Total**	697	100.0	1127	100.0

*The transactions could have been recorded at one or more store postcodes.
[t] Purchases at Northern Ireland are excluded due to small numbers (n<5)

However, there were no significant differences between the Index of Multiple Deprivation (IMD) quintiles recorded for store locations where overall purchases took place between cases and controls, $X^2(4, 1500) = 1.67$, p=0.79 (See Table 4). Among cases, 105 transactions (20.5%) took place in stores located in the most deprived quintile (1), comparison to 188 purchases (19.0%) among controls. The lowest numbers of transactions were recorded in stores located in the least deprived quintile for both cases (n= 67, 13.1%) and controls (n=124, 12.5%).

Table 4. The number of transactions recorded on loyalty cards among participants based on the Index of Multiple Deprivation (IMD) Quintiles

Index of Multiple Deprivation Quintile	Transactions			
	Cases		Controls	
	N *	%	N*	%
**1 (Most Deprived)**	105	20.5	188	19.0

For peer review only - http://bmjopen.bmj.com/site/about/guidelines.xhtml

**2**	129	23.5	261	26.4
**3**	121	23.7	231	23.4
**4**	98	19.2	185	18.7
**5 (Least Deprived)**	65	13.1	124	12.5
**Total[1]**	511	100.0	989	100.0

1. No postcode-IMD conversions were recorded for the store postcodes located in Wales and Scotland.

Cost per control participant recruitment

Most controls were recruited through paid social media advertisement (n=249, 58.3%), costing on average £15 per participant compared to opportunistic methods which were not paid (n=178, See Table 5). About 31% (n=133) of control participants were recruited opportunistically during additional research that investigated attitudes towards CLOCS and willingness to take part, but their recruitment pathway was not recorded at the time (missing data, n=131, 31%). The cost per participant for those non-targeted studies was an average of £5.

Table 5: Where control participants reported they heard about the study and cost of recruitment per participant from that source.

Recruitment source	N	%	Cost per participant (GBP)
From a patient	4	0.9	0.00
HSR Email	4	0.9	0.00
Twitter	2	0.5	0.00
Facebook	249	58.3	15.00
Instagram	3	0.7	0.00
Press	1	0.2	0.00
Word of Mouth	23	5.4	0.00
VOICE	8	1.9	0.00
Missing*	133	31.1	5.00

For peer review only - http://bmjopen.bmj.com/site/about/guidelines.xhtml

**Total**	427	100.0	

*Number of participants recruited prior to amendment to the research protocol in September 2020 that included a new item measuring the source of recruitment

Risk Factor Questionnaire Completion Rates

The majority of the items on the risk factor questionnaire were completed by both cases and controls, with the largest proportion of missing data observed for menopausal status among control participants (21.08%), followed by breastfeeding duration (18.50%), and BMI (6.79%). Both cases and controls had similar missing values for the number of full-term birth (4.40% vs. 6.09%), the number of non-full-term birth (6.59% vs 7.26%), and age at first birth (3.85% vs 4.22%). The distribution of the missing data is reported in Supplementary Table 1.

DISCUSSION

The participant recruitment and data retention in this observational case-control study demonstrate that this is a feasible method and is acceptable to the public to investigate individual transactional data for health research. This is a major achievement in social sciences and epidemiological research to further understand the impact of individual behaviours on health outcomes with robust and objective datasets. Furthermore, despite the challenges of the COVID-19 pandemic, when recruitment was finalised in 31st January 2022, the study was only 73 participants short of the initial target to recruit 500 control participants. CLOCS recruited approximately 10 cases and 25 controls per month over the course of 17 active recruitment months. Most control participants were willing to take part (n=427) and provided their consent for the researchers to request their transactional loyalty card history from the high street retailers. However, the researchers were unable to process over two thirds of the control participants consent due to the lack of identity verification

For peer review only - http://bmjopen.bmj.com/site/about/guidelines.xhtml

details. Furthermore, about 25% and 14% of cases and controls who provided information, including the name on the card and the long digit card number, did not match with the retailers' records, resulting in only 173 cases and 138 controls included in the final transactional data analyses.

The representativeness of the study population was assessed with the comparison of the population characteristics as well as the comparison of the population based on the postcode level characteristics of the store locations recorded on the loyalty cards. A key outcome is younger individuals without ovarian cancer were more willing to take part than those who were 60 years and older, despite all age groups being targeted as part of the recruitment process. This sampling bias is commonly observed in many survey studies with broad inclusion criteria for those who are eligible to take part [6] and unable to reach the sample size without careful targeting. On the other hand, CLOCS case participants were representative of the age distribution of ovarian cancer patients in the UK [3] and were recruited across 12 research sites. The imbalance in age between cases and controls is a potential barrier for 1:1 case and control age-matched analyses as the majority of ovarian cancer patients were aged 60 years old and above. Therefore, it is proposed that 2:1 age matching with replacement is used adjusting for number of people in the household, and ovarian cancer risk factors that are found to be significantly associated with ovarian cancer risk among CLOCS participants. This solution to case-control matching is in line with other case-control studies with insufficient control participants to match each case [13].

There were also some contextual barriers as an outcome of the COVID-19 pandemic to be considered for future research. It is suggested that the way the UK government collected data to inform public health strategies may have had a negative influence on people's

For peer review only - http://bmjopen.bmj.com/site/about/guidelines.xhtml

willingness to take part in studies like CLOCS. In July 2020, after the first lockdown restrictions were eased in the UK [14], we carried out a population-based survey in England asking individuals their willingness to share commercial data including shopping, internet searches, wearable devices, and social media data for health research (Hirst, Flanagan et al, unpublished data). A key finding of this study was that two-thirds of the population were willing to share their commercial data with academics for health research, and willingness to share data reduced with the participants' ages. Also, only half of the respondents were happy to share shopping data (51.8 %) although it was more acceptable compared to internet searches (35.2%), smart phone applications (32%), wearable devices (31.8%), and social media data (30.5%) (Hirst, Flanagan et al, unpublished data). These findings are reflective of the reduction in public trust in the government and the negative perceptions towards the contact tracing app that collected personal information using mobile phone data [15,16].

Although General Data Protection Regulation (GDPR) now allows individuals to request their data from one data controller and share with third parties [9], transactional data is considered sensitive data and is, therefore, subject to strict data security measures by data controllers. Although ID verification is not required by all organisations, it is recommended for researchers to verify the authenticity of the participants so that data of individuals are not shared with third party organisations unlawfully. It is possible that the process of requesting ID verification details in CLOCS may not have been as clear to the participants who agreed to take part in the study resulting in drop-outs. Previous studies have shown that individuals only read part of the study information and the consent form [17, 18] which may explain why 14 control participants withdrew after they were asked to provide ID

For peer review only

verification details and a majority did not respond to our email reminders. A potential solution to reduce the number of drop-outs could be to use dynamic consent processes and two-step authentication processes that are commonly used in other secure access scenarios such as online banking and shopping [19]. Dynamic consent gives the user more control over when they want to share their data, how they want to share it, and allows researchers to amend the consent if necessary to add new variables and datasets [19].

In conclusion, this paper demonstrates the feasibility outcomes of CLOCS and discusses how to resolve the issues in the future to maximise participation and data retention rates. Specifically, it shows that although the general public is amenable to taking part and providing consent for their data to be shared with researchers, due to robust application of the data sharing principles based on GDPR and Data Security Act 2018 by the researchers and the high street retailers, only a third of the dataset was included for the control participants. The contextual and procedural barriers as well as the public acceptability of data sharing should be considered in the design of future studies to ensure that individuals' contributions to health research with transactional data is successful.

For peer review only

ACKNOWLEDGEMENTS

The authors would like to acknowledge all of the participants in CLOCS, the participating retailers, and the NHS sites that recruited ovarian cancer patients to this study. We would like to acknowledge Deborah Tanner and Fiona Murphy who have acted as ovarian cancer patient representatives since the inception of the CLOCS project. This study was funded by Cancer Research UK Early Detection and Diagnosis project grant (C38463/A26726) with additional support from the Cancer Research UK Imperial Centre, the National Institute for

Health Research Imperial Biomedical Research Centre and the Ovarian Cancer Action Research Centre. The authors have no conflicts of interest to declare.

CONFLICT OF INTEREST STATEMENT

The authors declare no conflicts of interest for this study.

AUTHOR CONTRIBUTIONS

JMF, YH, HRB conceived the study. HRB and YH conducted all statistical analyses. HRB, JMF and YH reviewed the results. EJ provided expertise in information governance and data management. HRB wrote the first draft of the manuscript. YH, JMF, SS and MCH commented and reviewed the final draft of the manuscript. All authors agree on the final version of the manuscript.

DATA SHARING STATEMENT

Access to all CLOCS purchase history data collected from the retailers is restricted due to the data sharing agreements in place and the sensitive nature of the data. We, however, welcome other researchers to contact us for collaborations. The PI and research team will review each proposal and, if approved, the analysis will be conducted by a member of the CLOCS team.

ETHICAL APPROVAL

CLOCS was reviewed and approved by the North West-Greater Manchester South Research Ethics Committee (19/NW/0427). Explicit consent for the research team to request loyalty card data from participating retailers and to take part in the study was given from all participants.

For peer review only - http://bmjopen.bmj.com/site/about/guidelines.xhtml

TRANSPARENCY STATEMENT

All authors confirm that the manuscript is an honest, accurate, and transparent account of the study, and no important aspects of the study have been omitted.

FUNDING

The Cancer Loyalty Card Study (CLOCS) is funded by Cancer Research UK (CRUK) Early Detection Project Grant (C38463/A26726). YH and HRB are funded by CRUK.

For peer review only

REFERENCES

1. Smittenaar, C., Petersen, K., Stewart, K. *et al.* Cancer incidence and mortality projections in the UK until 2035. *Br J Cancer* 2016. **115,** 1147–1155. https://doi.org/10.1038/bjc.2016.304
2. Funston G, Hamilton W, Abel G, Crosbie EJ, Rous B, Walter FM. The diagnostic performance of CA125 for the detection of ovarian and non-ovarian cancer in primary care: A population-based cohort study. PLoS Med. 2020 Oct 28;17(10):e1003295. doi: 10.1371/journal.pmed.1003295

For peer review only - http://bmjopen.bmj.com/site/about/guidelines.xhtml

3. Cancer Research UK. 2022. *Ovarian cancer statistics*. [online] Available at: <https://www.cancerresearchuk.org/health-professional/cancer-statistics/statistics-by-cancer-type/ovarian-cancer> [Accessed 10 May 2022].

4. Brewer HR, Hirst Y, Sundar S, et al. Cancer Loyalty Card Study (CLOCS): protocol for an observational case-control study focusing on the patient interval in ovarian cancer diagnosis. BMJ Open Published Online First: 2020. doi:10.1136/bmjopen-2020-037459

5. Flanagan J, Skrobanski H, Shi X, et al. Self-Care Behaviors of Ovarian Cancer Patients Before Their Diagnosis: Proof-of-Concept Study. JMIR Cancer 2019;5:e10447. doi:10.2196/CANCER.10447

6. Grimes DA, Schulz KF. Compared to what? Finding controls for case-control studies. The Lancet. 2005 Apr 16;365(9468):1429-33.

7. Bernstein L. Control recruitment in population-based case-control studies. Epidemiology. 2006 May 1;17(3):255-7.

8. VOICE. 2022. *Imperial College London - VOICE*. [online] Available at: <https://www.voice-global.org/about/imperial-college-london/.> [Accessed 10 May 2022].

9. GDPRhub. 2022. *Article 15 GDPR*. [online] Available at: <https://gdprhub.eu/Article_15_GDPR> [Accessed 10 May 2022].

10. Heckathorn DD, Broadhead RS, Sergeyev B. A methodology for reducing respondent duplication and impersonation in samples of hidden populations. Journal of Drug Issues. 2001 Apr;31(2):543-64.

For peer review only

For peer review only - http://bmjopen.bmj.com/site/about/guidelines.xhtml

11. Imd-by-postcode.opendatacommunities.org. 2022. *English indices of deprivation 2019: Postcode Lookup*. [online] Available at: <https://imd-by-postcode.opendatacommunities.org/imd/2019> [Accessed 10 May 2022].

12. Bath.ac.uk. 2022. [online] Available at: <https://www.bath.ac.uk/publications/undergraduate-bursaries-and-scholarships-terms-and-conditions-2020-21/attachments/Indices-of-Multiple-Deprivation-Lookup-Guide.pdf> [Accessed 10 May 2022].

13. Pearce N. Analysis of matched case-control studies. BMJ. 2016 Feb 25;352.

14. Instituteforgovernment.org.uk. 2022. [online] Available at: <https://www.instituteforgovernment.org.uk/charts/uk-government-coronavirus-lockdowns> [Accessed 10 May 2022].

15. How has COVID-19 affected trust in scientists? [Internet]. IPSOS MORI; 2022 [cited 10 May 2022]. Available from: https://www.ukri.org/wp-content/uploads/2020/09/UKRI-271020-COVID-19-Trust-Tracker.pdf

16. Samuel G, Roberts SL, Fiske A, Lucivero F, McLennan S, Phillips A, Hayes S, Johnson SB. COVID-19 contact tracing apps: UK public perceptions. Critical Public Health. 2022 Jan 1;32(1):31-43.

17. Flory J, Emanuel E. Interventions to improve research participants' understanding in informed consent for research: a systematic review. Jama. 2004 Oct 6;292(13):1593-601.

18. D'Abramo F, Schildmann J, Vollmann J. Research participants' perceptions and views on consent for biobank research: a review of empirical data and ethical analysis. BMC Med Ethics. 2015;16:60.

For peer review only - http://bmjopen.bmj.com/site/about/guidelines.xhtml

19. Budin-Ljøsne I, Teare HJ, Kaye J, Beck S, Bentzen HB, Caenazzo L, Collett C, D'Abramo F, Felzmann H, Finlay T, Javaid MK. Dynamic consent: a potential solution to some of the challenges of modern biomedical research. BMC medical ethics. 2017 Dec;18(1):1-0.

For peer review only

Figure 1. CLOCS recruitment timeline for cases and controls.

Figure 2. Timeline of when CLOCS case participants were diagnosed with ovarian cancer by quarter (Q) and year.

Figure 3. CLOCS Consort Diagram detailing participant recruitment

For peer review only - http://bmjopen.bmj.com/site/about/guidelines.xhtml

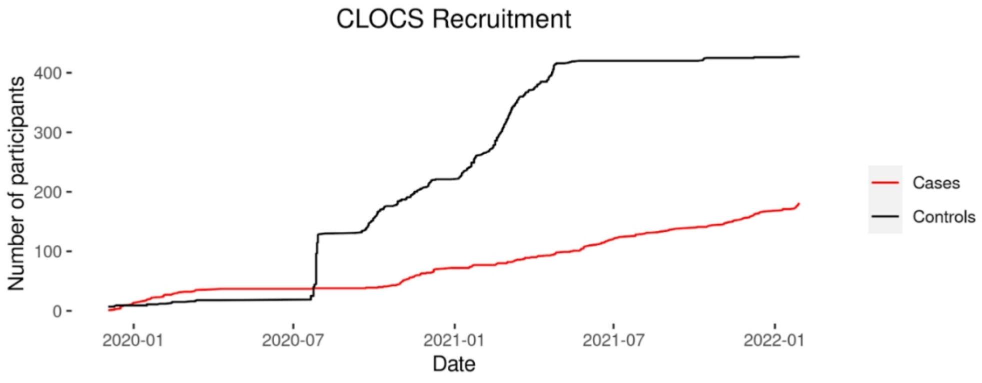

Figure 1

159x61mm (220 x 220 DPI)

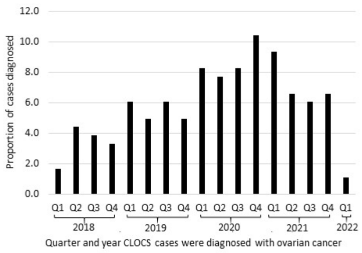

Figure 2

338x190mm (96 x 96 DPI)

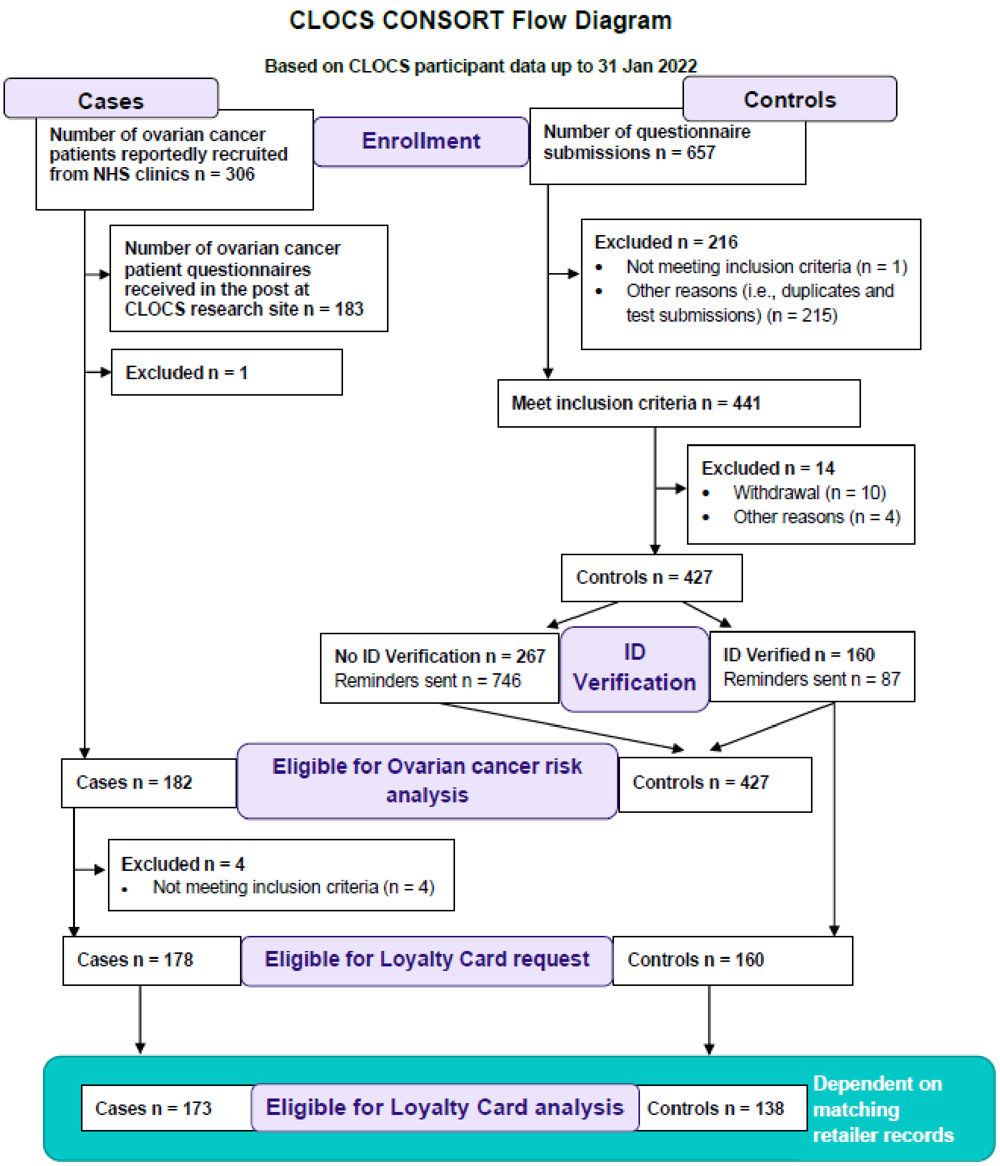

Figure 3

174x203mm (96 x 96 DPI)

Cancer Loyalty Card Study (CLOCS): feasibility outcomes for an observational case-control study focusing on the patient interval in ovarian cancer

Hannah R. Brewer[1], Marc Chadeau-Hyam[2], Eric Johnson[2], Sudha Sundar[3], James M. Flanagan[1*] and Yasemin Hirst[4]

For peer review only

SUPPLEMENTARY MATERIAL

Supplementary Table 1: Ovarian Cancer Risk questionnaire item distribution and missing data

Risk factor questionnaire items	Cases		Controls	
	N	%	N	%
**Age (years)**	182	100.00	422	98.83
Missing	*0*	*0.00*	*5*	1.17
**Age at menarche (years)**				
08-11	38	20.88	95	22.25
12	43	23.63	89	20.84
13	39	21.43	101	23.65
14-19	57	31.32	138	32.32
Missing	*5*	2.75	*4*	0.94
**OC use**				
Never	43	23.63	50	11.71
Ever	138	75.82	377	88.29
Missing	*1*	0.55	*0*	0.00
**Duration of OC use (years)**				
0	52	28.57	56	13.11
1-5	59	32.42	140	32.79
>5	71	39.01	231	54.10
Missing	*0*	0.00	*0*	0.00
**Parity**				
Never	42	23.10	109	25.53
Ever	139	76.40	316	74.00
Missing	*1*	*0.50*	*2*	0.47
**No. full-term births**				

For peer review only - http://bmjopen.bmj.com/site/about/guidelines.xhtml

0	55	30.22	138	32.32
1	29	15.93	70	16.39
2	59	32.42	129	30.21
≥3	31	17.03	64	14.99
Missing	*8*	4.40	*26*	6.09
**No. non full-term births**				
0	125	68.68	244	57.14
1	29	15.93	97	22.72
2	10	5.49	29	6.79
>=3	6	3.30	26	6.09
Missing	*12*	6.59	*31*	7.26
**Age last birth**				
Never	49	26.92	128	29.98
<=25	25	13.74	45	10.54
26-30	44	24.18	83	19.44
>=31	57	31.32	153	35.83
Missing	*7*	3.85	*18*	4.22
**Breastfeeding**				
Never	94	51.65	202	47.31
Ever	87	47.80	220	51.52
Missing	*1*	0.55	*5*	1.17
**Breastfeeding duration (months)**				
Never	96	52.75	144	33.72
01-6	43	23.63	92	21.55
07-12	15	8.24	33	7.73
>12	27	14.84	79	18.50
Missing	*0*	0.00	*79*	18.50
**Tubal ligation**				
Never	154	84.62	376	88.06
Ever	22	12.09	51	11.94
Missing	*6*	3.30	*0*	0.00
**Endometriosis**				
Not diagnosed	170	93.41	393	92.04
Diagnosed	12	6.59	34	7.96
Missing	*0*	0.00	*0*	0.00
**Family history of breast cancer (first-degree relative)**				
None	150	82.42	366	85.71
Yes	31	17.03	60	14.05
Missing	*1*	0.55	*1*	0.23
**Family history of ovarian cancer (first-degree relative)**				
None	166	91.21	379	88.76
Yes	16	8.79	46	10.77

For peer review only

Missing	*0*	0.00	*2*	0.47
**BMI**				
Underweight (BMI < 18.5)	5	2.75	4	0.94
Normal (18.5 – 25)	75	41.21	138	32.32
Overweight (25-30)	50	27.47	131	30.68
Obese (>30)	44	24.18	125	29.27
Missing	*8*	4.40	*29*	6.79
**Aspirin use**				
Irregular or no use	159	87.36	390	91.33
Regular user of low-dose aspirin	18	9.89	28	6.56
Regular user of high-dose aspirin	3	1.65	1	0.23
Missing	*2*	1.10	*8*	1.87
**Menopausal status**				
Premenopausal	8	4.40	106	24.82
Postmenopausal	163	89.56	231	54.10
Missing	*11*	6.04	*90*	21.08
**Hysterectomy**				
Never	45	24.73	399	93.44
Ever	133	73.08	26	6.09
Missing	*4*	2.20	*2*	0.47
**HRT use**				
Never	123	67.58	343	80.33
Ever	58	31.87	84	19.67
Missing	*1*	0.55	*0*	0.00
**Duration of HRT use (years)**				
0	124	68.13	344	80.56
1-5	31	17.03	58	13.58
>5	27	14.84	21	4.92
Missing	*0*	0.00	*3*	0.70
**Smoking**				
Never	102	56.04	261	61.12
Ever	80	43.96	165	38.64
Missing	*0*	0.00	*1*	0.23
**Duration of Smoking (years)**				
0	102	56.04	261	61.12
1-10	27	14.84	55	12.88
>10-20	21	11.54	47	11.01
>20	32	17.58	60	14.05
Missing	*0*	0.00	*4*	0.94
**Vaping**				
Never	173	95.05	394	92.27
Ever	9	4.95	32	7.49
Missing	*0*	0.00	*1*	0.23

For peer review only - http://bmjopen.bmj.com/site/about/guidelines.xhtml

Duration of Vaping (years)				
0	173	95.05	394	92.27
1-5	7	3.85	27	6.32
>5	2	1.10	4	0.94
Missing	*0*	0.00	*2*	0.47

For peer review only

STROBE Statement—Checklist of items that should be included in reports of ***case-control studies***

	Item No	Recommendation	Page No
**Title and abstract**	1	(*a*) Indicate the study's design with a commonly used term in the title or the abstract	2
		(*b*) Provide in the abstract an informative and balanced summary of what was done and what was found	3
**Introduction**			
Background/rationale	2	Explain the scientific background and rationale for the investigation being reported	4
Objectives	3	State specific objectives, including any prespecified hypotheses	4
**Methods**			
Study design	4	Present key elements of study design early in the paper	4
Setting	5	Describe the setting, locations, and relevant dates, including periods of recruitment, exposure, follow-up, and data collection	4-5
Participants	6	(*a*) Give the eligibility criteria, and the sources and methods of case ascertainment and control selection. Give the rationale for the choice of cases and controls	5
		(*b*) For matched studies, give matching criteria and the number of controls per case	7
Variables	7	Clearly define all outcomes, exposures, predictors, potential confounders, and effect modifiers. Give diagnostic criteria, if applicable	6-7
Data sources/ measurement	8*	For each variable of interest, give sources of data and details of methods of assessment (measurement). Describe comparability of assessment methods if there is more than one group	7
Bias	9	Describe any efforts to address potential sources of bias	7
Study size	10	Explain how the study size was arrived at	Published (Brewer et al BMJ Open 2020)
Quantitative variables	11	Explain how quantitative variables were handled in the analyses. If applicable, describe which groupings were chosen and why	7
Statistical methods	12	(*a*) Describe all statistical methods, including those used to control for confounding	6-7
		(*b*) Describe any methods used to examine subgroups and interactions	7
		(*c*) Explain how missing data were addressed	6
		(*d*) If applicable, explain how matching of cases and controls was addressed	7
		(*e*) Describe any sensitivity analyses	7
**Results**			
Participants	13*	(a) Report numbers of individuals at each stage of study—eg numbers potentially eligible, examined for eligibility, confirmed eligible, included in the study, completing follow-up, and analysed	Figure 1 and page 8
		(b) Give reasons for non-participation at each stage	8
		(c) Consider use of a flow diagram	Figure 1
Descriptive data	14*	(a) Give characteristics of study participants (eg demographic, clinical,	Table 1, page

For peer review only - http://bmjopen.bmj.com/site/about/guidelines.xhtml

		social) and information on exposures and potential confounders	20
		(b) Indicate number of participants with missing data for each variable of interest	Supplementary Tables
Outcome data	15*	Report numbers in each exposure category, or summary measures of exposure	Supplementary Tables

For peer review only

For peer review only - http://bmjopen.bmj.com/site/about/guidelines.xhtml

Main results	16	(*a*) Give unadjusted estimates and, if applicable, confounder-adjusted estimates and their precision (eg, 95% confidence interval). Make clear which confounders were adjusted for and why they were included	9
		(*b*) Report category boundaries when continuous variables were categorized	NA
		(*c*) If relevant, consider translating estimates of relative risk into absolute risk for a meaningful time period	NA
Other analyses	17	Report other analyses done—eg analyses of subgroups and interactions, and sensitivity analyses	9-10
**Discussion**			
Key results	18	Summarise key results with reference to study objectives	10
Limitations	19	Discuss limitations of the study, taking into account sources of potential bias or imprecision. Discuss both direction and magnitude of any potential bias	12
Interpretation	20	Give a cautious overall interpretation of results considering objectives, limitations, multiplicity of analyses, results from similar studies, and other relevant evidence	13
Generalisability	21	Discuss the generalisability (external validity) of the study results	13
**Other information**			
Funding	22	Give the source of funding and the role of the funders for the present study and, if applicable, for the original study on which the present article is based	16

*Give information separately for cases and controls.

Note: An Explanation and Elaboration article discusses each checklist item and gives methodological background and published examples of transparent reporting. The STROBE checklist is best used in conjunction with this article (freely available on the Web sites of PLoS Medicine at http://www.plosmedicine.org/, Annals of Internal Medicine at http://www.annals.org/, and Epidemiology at http://www.epidem.com/). Information on the STROBE Initiative is available at http://www.strobe-statement.org.

For peer review only

BMJ Open

Cancer Loyalty Card Study (CLOCS): feasibility outcomes for an observational case-control study focusing on the patient interval in ovarian cancer

Journal:	*BMJ Open*
Manuscript ID	bmjopen-2022-066022.R1
Article Type:	Original research
Date Submitted by the Author:	02-Mar-2023
Complete List of Authors:	Brewer, Hannah; Imperial College London, Surgery and Cancer<br>Chadeau-Hyam, Marc; Imperial College London, MRC Centre for Environment and Health; Imperial College London, Epidemiology and Biostatistics, School of Public Health<br>Johnson, Eric; Imperial College London<br>Sundar, Sudha; University of Birmingham, Cancer Sciences<br>Flanagan, James; Imperial College London, Department of Surgery and Cancer<br>Hirst, Yasemin; University College London, Epidemiology and Public Health
**Primary Subject Heading**:	Oncology
Secondary Subject Heading:	Epidemiology, Health informatics, Research methods
Keywords:	Health informatics < BIOTECHNOLOGY & BIOINFORMATICS, Epidemiology < ONCOLOGY, Gynaecological oncology < ONCOLOGY

SCHOLARONE™
Manuscripts

I, the Submitting Author has the right to grant and does grant on behalf of all authors of the Work (as defined in the below author licence), an exclusive licence and/or a non-exclusive licence for contributions from authors who are: i) UK Crown employees; ii) where BMJ has agreed a CC-BY licence shall apply, and/or iii) in accordance with the terms applicable for US Federal Government officers or employees acting as part of their official duties; on a worldwide, perpetual, irrevocable, royalty-free basis to BMJ Publishing Group Ltd ("BMJ") its licensees and where the relevant Journal is co-owned by BMJ to the co-owners of the Journal, to publish the Work in this journal and any other BMJ products and to exploit all rights, as set out in our licence.

The Submitting Author accepts and understands that any supply made under these terms is made by BMJ to the Submitting Author unless you are acting as an employee on behalf of your employer or a postgraduate student of an affiliated institution which is paying any applicable article publishing charge ("APC") for Open Access articles. Where the Submitting Author wishes to make the Work available on an Open Access basis (and intends to pay the relevant APC), the terms of reuse of such Open Access shall be governed by a Creative Commons licence – details of these licences and which Creative Commons licence will apply to this Work are set out in our licence referred to above.

Other than as permitted in any relevant BMJ Author's Self Archiving Policies, I confirm this Work has not been accepted for publication elsewhere, is not being considered for publication elsewhere and does not duplicate material already published. I confirm all authors consent to publication of this Work and authorise the granting of this licence.

For peer review only

Cancer Loyalty Card Study (CLOCS): feasibility outcomes for an observational case-control study focusing on the patient interval in ovarian cancer

Hannah R. Brewer[1], Marc Chadeau-Hyam[2], Eric Johnson[2], Sudha Sundar[3], James M. Flanagan[1*] and Yasemin Hirst[4]

1. Division of Cancer, Department of Surgery and Cancer, Imperial College London, London

2. School of Public Health, Imperial College London, London

3. Institute of Cancer and Genomic Sciences, University of Birmingham, Birmingham

4. Department of Behavioural Science and Health, University College London, London

***Address for correspondence:**

Dr. James M. Flanagan

Division of Cancer, Department of Surgery and Cancer,

Faculty of Medicine, Imperial College London

4th Floor IRDB, Hammersmith Campus, Du Cane Road, London W12 0NN

Tel: 020 75942127; email: j.flanagan@imperial.ac.uk;

Word Count

Body: 4141

Abstract: 289

For peer review only - http://bmjopen.bmj.com/site/about/guidelines.xhtml

ABSTRACT

Objectives: Ovarian cancer symptoms are often non-specific and can be normalised before patients seek medical help. The Cancer Loyalty Card Study (CLOCS) investigated self-management behaviours of ovarian cancer patients prior to their diagnosis using loyalty card data collected by two United Kingdom (UK)-based high street retailers. Here, we discuss the feasibility outcomes for this novel research.

Design: Observational case-control study.

Setting: Control participants were invited to the study using social media and other sources from the general public. Once consented, control participants were required to submit proof of identification (ID) for their loyalty card data to be shared. Cases were identified using unique National Health Service (NHS) numbers (a proxy for ID) and were recruited through 12 NHS tertiary care clinics.

Participants: Women in the UK, 18 years or older, with at least one of the participating high street retailers' loyalty cards. Those with an ovarian cancer diagnosis within two years of recruitment were considered cases, and those without an ovarian cancer diagnosis were considered controls.

Primary Outcome Measures: Recruitment rates, demographics of participants and identification of any barriers to recruitment.

Results: In total, 182 cases and 427 controls were recruited with significant differences by age, number of people in participants' households and the geographical region in the UK. However, only 37% (n=160/427) of control participants provided sufficient ID details and 81% (130/160) matched retailers' records. The majority of the participants provided complete responses to the 24-Item Ovarian Risk Questionnaire.

Conclusions: Our findings show that recruitment to a study aiming to understand self-care behaviours using loyalty card data is challenging but feasible. The general public were willing to share their data for health research. Barriers in data sharing mechanisms need to be addressed to maximise participant retention.

Trial registration: ISRCTN 14897082, CPMS 43323, NCT03994653.

For peer review only

STRENGTHS AND LIMITATIONS OF THIS STUDY

Main Strengths:

- This study is the first of its kind to investigate transactional data for cancer outcomes.
- It has established data governance structures and methods to comply with GDPR and privacy policies for using retailer transactional data.
- This study provides a pre-existing prospectively collected data set free from recall biases which is a common bias in case-control studies.

Main Limitations:

- Recruitment happened during the COVID-19 pandemic, which affected recruitment rates, particularly for cases who were recruited through NHS clinics
- Increasing age was negatively associated with participation rates, particularly in the control population, requiring targeted recruitment to older participants.

KEYWORDS:

Observational case control study; feasibility ; recruitment ; transactional data ; cancer ; diagnosis

For peer review only

INTRODUCTION

Approximately 7500 new cases of ovarian cancer are diagnosed in the United Kingdom (UK) each year, and it is estimated that ovarian cancer incidence rates will rise by 15% in the UK between 2014 and 2035 [1]. Unfortunately, 23% of ovarian cancer patients in 2018 were diagnosed at Stage IV [2] when treatment options are less than optimal for survival. Late-stage ovarian cancer diagnosis is often associated with delayed patient presentation and a longer diagnostic interval due to non-specific cancer symptoms (e.g., feeling bloated, indigestion, feeling full, abdominal pain). While there has been progress in efforts to reduce diagnostic delays using routine blood tests (i.e., CA125) [3-5] there is little evidence focusing on women's self-care behaviours associated with managing non-specific symptoms. It has been demonstrated that population screening of asymptomatic women is not effective in reducing mortality from the disease and, therefore, is not recommended in the UK [6]. Screening in the symptomatic population may prove more effective [7], however, the main barrier to earlier diagnosis remains a lack of awareness of the vague non-specific symptoms of ovarian cancer [8].

The Cancer Loyalty Card Study (CLOCS) investigated the period between the presentation of the first symptom(s)/sign(s) leading up to the first presentation at health care providers (i.e., the patient interval) of ovarian cancer patients to better understand self-care behaviours using past purchase information collected through loyalty cards at two high street retailers in the UK [9]. This project aimed to better understand whether self-care behaviours can be detected at an early stage and facilitate earlier presentation to healthcare providers, and the main results have been reported elsewhere [10]. We have previously reported in a proof-of-concept study that loyalty card data may be used to identify an increase in

For peer review only - http://bmjopen.bmj.com/site/about/guidelines.xhtml

painkiller and indigestion medication purchases among ovarian cancer patients [11]. The proof-of-concept study warranted testing this hypothesis in a large-scale case-control study design aiming to include 500 ovarian cancer patients (cases) and 500 individuals without an ovarian cancer diagnosis (controls) [9].

Flanagan et al demonstrated the key information governance and ethical considerations of recruiting participants to a project that is requesting consent for transactional data to be used in health research [11]. These included separate consent forms for the study and data requests for each retailer, clear communication with potential participants about which information is being collected, stored, how it is being used, how long, and data breach risks, if any. Loyalty card ownership was also identified as a logistical barrier. Thus, ownership was recommended as a prerequisite of participation in the study to avoid data loss or invalidated consents. In terms of data reliability among cases, it was also recommended that patients should be recruited to the study close to their initial diagnosis for researchers to have a retrospective purchase history capturing self-care behaviours before ovarian cancer diagnosis.

Furthermore, recruitment and participant retention to case-control studies are not without challenges; they have often been criticised for potential selection bias and representativeness of control participants against case participants [12]. Traditional recruitment strategies often involve recruitment through healthcare organisations, random digit dialling and postal invitations, recruitment via family and friends of patients [13]. In CLOCS, due to low prevalence of ovarian cancer in the UK, a multi-site recruitment approach for cases in the UK was proposed with social media recruitment of controls to minimise selection bias and maximise representativeness [9]. However, while patients' willingness to take part may be higher for cancer research, it was also deemed necessary to improve

For peer review only - http://bmjopen.bmj.com/site/about/guidelines.xhtml

public acceptability among potential control participants by raising awareness and improving understanding using an accessible communication format, i.e., a tailored website (www.clocsproject.org.uk).

The purpose of this paper is to present the feasibility outcomes of CLOCS which aimed to recruit 1000 women with and without ovarian cancer in the UK, describe feasibility of a large-scale case-control study by assessing participant rates, characteristics and any possible changes to the study design that could optimise data collection in the future. In particular, we discuss the barriers and facilitators of recruiting control participants to a case-control study through social media platforms and case participants using ovarian cancer patient clinics in National Health Service (NHS) trusts in light of the COVID-19 pandemic.

METHODS

Design

The aims, design, and results of CLOCS have previously been reported [9,10]. CLOCS is an observational case-control study where participants are women in the UK, aged 18 years or older, who hold a loyalty card at either or both participating high street retailers (HSR1 and HSR2). Participants with a diagnosis of ovarian cancer were considered cases, and those without a previous ovarian cancer diagnosis were considered controls.

Setting/s

Cases were recruited from 12 NHS clinic sites in England, Wales, and Scotland. Eligible patients were identified by each site's recruitment team and presented with information in person about the study in paper format. If they decided to take part, participants were given

For peer review only - http://bmjopen.bmj.com/site/about/guidelines.xhtml

the consent form, a risk factor questionnaire, and a clinical questionnaire (completed by their clinical care team) to either complete in the clinic or take with them to complete at another time (Supplementary Material 1). Completed forms were then posted to the CLOCS main research site.

In response to the COVID-19 pandemic, an amendment was submitted and approved by the NHS Research Ethics Committee (20 September 2020, 19/NW.0427-SA1) to allow clinics to recruit eligible ovarian cancer patients over the phone, post them the study documents to complete where they were shielding, and post completed forms to the main research site.

Controls were recruited primarily using paid/non-paid web-based methods, snowball sampling, a pilot targeted email campaign from one of the participating high street retailers to its users, and word of mouth. These methods included paid Facebook advertising, distribution of study blogs on the study website, study twitter (@CLOCS_Imperial), VOICE Imperial College London [14], and widely distributed webinars that aimed to address barriers in CLOCS participation. In October 2021, a pilot email was sent to 1053 cardholders from one of the retailers to test the impact of sending invitations for CLOCS and measure the response rate to invitations.

Procedures

Consent + Questionnaire Completion (Step 1)

Each potential participant was provided with a detailed information sheet and privacy policy to facilitate informed decision making. This was followed by study consent, the study questionnaire, and a clinical questionnaire for cases. The CLOCS participant consent form

For peer review only - http://bmjopen.bmj.com/site/about/guidelines.xhtml

consisted of two sections. In the first section, participants consented to take part in the study which provided information on data retention, how data is being handled for the study, how long, and how to withdraw. In the second section, participants provided consent for the research team to request their purchase history from the participating retailer(s) using the exact numbers and names on their loyalty cards. Contact details were collected to clarify loyalty card details in the event their details did not match the retailers' records. Participants were given the option to consent to be re-contacted to take part in future related studies.

Case participants completed paper-based forms which were then posted back to the research team for secure data entry. Control participants were asked to complete a web-based secure study form including the information sheet, the consent form and risk factor questionnaire on the study's website (www.clocsproject.org.uk)

Substantial Amendment - ID verification (Step 2)

In September 2020, the CLOCS research team received approval from the North West - Greater Manchester South Research Ethics Committee (dated 20 September 2020, 19/NW/0427-SA1) to request identity verification details including a visual proof of ID (e.g., passport), and a proof of address (e.g., utility bill) from participants. This was a development during the research recruitment process before data sharing agreements with HSR1 and HSR2 were established. Thus, the aim of the ID verification step was to ensure that those participating in the study were not completing the forms on behalf of someone else and could be verified against the high street retailer's records. Therefore, control participants who took part in the study online were asked to provide a copy of a photo ID and proof of

For peer review only - http://bmjopen.bmj.com/site/about/guidelines.xhtml

address, using a unique and secure weblink, after Step 1 was completed. If they did not complete Step 2 at the time of the participation, they were sent a maximum of three reminder emails, which included their unique upload link. All ID verification documents were encrypted using private and public keys and kept until data requests from the retailers were completed or when the study was complete, whichever came first. Control participants who took part prior to September 2020 were sent unique secure links to consider uploading this information so that the research team could request their purchase history data from the retailers. If they did not upload the documents after their reminders, no requests were made from the retailers on their behalf. Identification of cases in NHS clinics using unique NHS numbers was sufficient for ID verification. Details of the transfer of participant purchase history between the research team and retailers are described in the protocol [9].

Participants who completed the consent form and risk factor questionnaire were eligible for the ovarian cancer risk analysis, regardless of whether they provided ID verification or their loyalty card details matched retailer records. We recontacted 39 participants to verify loyalty card details.

Recruitment Status

Recruitment was opened for ovarian patients from 1 November 2019 and for control participants from 1 July 2020. Recruitment was suspended from 31 January 2022 (the original planned closing date) pending funding decisions and was finally closed on 28 July 2022.

For peer review only - http://bmjopen.bmj.com/site/about/guidelines.xhtml

Patient and Public Involvement (PPI)

PPI informed the design and the recruitment strategies throughout CLOCS. We presented two public facing seminars in December 2020 aiming to engage with the wider audience about CLOCS and changes in the participation criteria using ID verification and in March 2021 during Ovarian Cancer Awareness month aiming to discuss the potential impact for ovarian cancer patients. Furthermore, we held three scientific meetings annually inviting academics, patient representatives, and participating high street retailers.

Feasibility outcomes measures

The following measures were collected and used to assess feasibility outcomes of CLOCS based on guidance on reporting feasibility outcomes and the reporting of observational studies in Epidemiology (STROBE).

Participant Descriptive Characteristics: Date of birth was collected and re-categorised in 10-year age groups for reporting. Other characteristics included ethnicity, the number of people in the participant's household (1 to ≥5 persons), self-reported loyalty card usage (not at all/not very often/sometimes/often/all the time/missing), loyalty card membership for the retailers (HSR1, HSR2, both and neither) included in the study, and family history of ovarian and/or breast cancer (Yes/No/Missing). We included 17 different ethnic backgrounds to be recorded, due to the small number participants in sub-populations, we categorise this as white/non-white/prefer not to say and missing.

The questionnaire was amended to include a question about whether participants had been diagnosed with COVID-19 (responses limited to the following: diagnosed and recovered,

For peer review only - http://bmjopen.bmj.com/site/about/guidelines.xhtml

diagnosed and still ill, suspected but not formally diagnosed/did not have COVID-19) at the time of the study participation.

Further data included the Index of Multiple Deprivation (IMD) quintiles based on the postcodes of the stores where purchases were made ranging from most deprived (1) to least deprived (5). The location where the purchases were made was included based on the nine UK Regions (East of England & Yorkshire and Humber, London, North East, North West, Midlands, Scotland, South East, South West, and Wales, excluding any purchases that were made in Northern Ireland due to small numbers [n<5]).

Ovarian Cancer Risk Questionnaire (24 Items)

Participants completed a short questionnaire about ovarian cancer risk factors including ethnicity, marital status, body mass index (BMI), age at menarche, menopausal status, age at menopause, parity, breastfeeding, hysterectomy, tubal ligation, cancer history, endometriosis, aspirin use, oral contraceptive (OC) use, hormone replacement therapy (HRT) use, family history of ovarian and breast cancers, vaping, and cigarette smoking. The questionnaire requested information about the symptoms experienced (if any) and number of visits to the general practitioner in the year leading up to participation for controls and cancer referral or diagnosis for cases. All responses were voluntary.

Participant Retention

Participant retention was measured at three stages. The first stage was the number of participants with a valid consent and completed ovarian cancer risk questionnaire. Participant retention was calculated based on withdrawals and valid consent (e.g. provided consent for retailers).

For peer review only - http://bmjopen.bmj.com/site/about/guidelines.xhtml

The second stage assessed the number of control participants with valid identity verification details. Identity verification was recorded as ID verified or not ID.

The last stage of retention was assessed based on the confirmation of the records with the retailers following transactional data requests. If participants' reported card details were the same as the retailers' and data was received, this was recorded as complete participation. If their reported card details did not match the retailers' records, we recorded these participants as unmatched.

Statistical Analysis

Participation and data retention was recorded in a CONSORT diagram. A chi-square test was used to assess the differences between cases and controls based on the following characteristics: age (10-year age-groups), ethnicity, number of people in the household, loyalty card ownership, and the location and IMD quintile of the stores where transactions took place. Each postcode for the stores where individuals purchased items were converted to the UK regions and IMD deciles using the English Indices of Deprivation 2019 [15]. These were then recoded into quintiles ranging from most deprived to least deprived [16]. If the sample size was less than 5, groups were merged together to ensure safe data reporting. Missing data and questionnaire completion rates were calculated.

RESULTS

Recruitment Timeline

The cumulative recruitment of CLOCS cases and controls is shown in Figure 1. CLOCS recruitment began in December 2019, and at the beginning of the COVID-19 pandemic in

For peer review only - http://bmjopen.bmj.com/site/about/guidelines.xhtml

the UK in March 2020, recruitment was paused to investigate the impact of the pandemic on recruitment while patients were not able to attend clinics.

Active control recruitment was also paused, however, opportunistic recruitment occurred through secondary sources. The changes were implemented, and patient recruitment resumed from September 2020. Facebook Advertising was used to recruit control participants from 17 September 2020 - 26 April 2021.

Participant Characteristics (included in ovarian cancer risk analysis)

The characteristics of CLOCS participants included in the final dataset for ovarian cancer risk analysis are shown in Table 1.

Table 1: Participant Characteristics

Characteristic	Cases (N= 182)		Controls (N=427)		
	N	%	N	%	*P*
**Age**					
18-39	6	3.6	61	16.2	<0.001
40-49	7	4.7	96	23.8	
50-59	32	21.3	125	31.8	
60-69	64	36.7	107	21.2	
≥70	73	33.7	33	5.9	
missing	0	0.0	5	1.2	
**Race and ethnicity**					
White	173	95.1	400	93.7	0.392
Non-white	8	4.4	11	2.6	
Prefer not to say	0	0.0	0	0.0	
Missing	1	0.5	16	3.7	
**Number of people in the household**					
1	35	22.2	45	14.5	<0.001
2	78	52.1	120	40.5	
3	17	11.8	62	20.9	

For peer review only - http://bmjopen.bmj.com/site/about/guidelines.xhtml

4	10	6.9	45	15.2	
≥5	2	1.4	24	8.1	
missing	8	5.6	2	0.7	
**Family history of breast or ovarian cancer**					
No	139	76.4	327	76.6	1.00
Yes	43	23.6	99	23.2	
Missing	0	0.0	1	0.2	
**Loyalty Card**					
HSR1 only	48	26.4	169	39.6	0.001
HSR2 only	44	24.2	116	27.2	
Both HSR1 and HSR2	86	47.3	142	33.3	
Neither	4	2.2	0	0.0	
**Loyalty Card Use**					
Not at all	1	0.5	2	0.5	0.449
Not very often	9	4.9	10	2.3	
Sometimes	18	9.9	42	9.8	
Often	42	23.1	117	27.4	
All the time	109	59.9	256	60.0	
Missing	3	1.6	0	0.0	

Cases

NHS recruitment sites reported that 306 patients were eligible and interested to take part in CLOCS. Of those, 183 patients (59%) returned their completed consent form and questionnaires. After exclusion due to insufficient consent, 182 were included in the final dataset for ovarian cancer risk analysis. About 70 % of cases who took part in CLOCS were diagnosed from January 2020 onward (Figure 2).

Most cases were 60 years or older (70.4%). The majority of the cases identified themselves as white ethnic background (95.1%), and the rest of the cases were grouped together as non-white ethnic background due to small numbers (4.4%). Over half of case participants reported living in a two-member household (52.1%), followed by 22.2% in a single-member

For peer review only

For peer review only - http://bmjopen.bmj.com/site/about/guidelines.xhtml

household, and 20.1% living in a household of three or more people. About 25% of cases had a family history of ovarian and/or breast cancer in first-degree, female relative.

Controls

In total, there were 657 online submissions of the secure online questionnaire (See Figure 2). Of those, 441 (61.7%) were considered valid control participants once duplicates and test submissions were removed. There were 14 participants who were excluded from the study; 10 withdrew from the study, and 4 were ineligible. Therefore, 427 control participants were included in the final dataset for ovarian cancer risk analysis.

Compared to case participants, controls were younger with a larger proportion in age groups 40-49 years (23.8%) and 50-59 years (31.8%). There were also significantly fewer control participants recruited in the older age groups 60-69 years (21.2%) and 70 years and older (5.9%) compared with case participants ($p<0.05$). Similar to cases, most controls reported living in a two-member household (40.5%), 14.5% in a single-member household, and 44.2% in a household with three or more individuals. Also, 76.6% of control participants reported having a first-degree, female relative with ovarian and/or breast cancer.

Participant Retention Rates

The recruitment pathways for cases and controls and how participants were retained within the study are reported in the CONSORT diagram in Figure 3.

While case participants did not require additional identity verification, there were four cases who did not provide loyalty card details, and therefore, 178 cases were eligible for data

For peer review only - http://bmjopen.bmj.com/site/about/guidelines.xhtml

requests from the retailers. In total 134 (73.6%) case participants were eligible for data requests from the HSR1 and 130 (71.4%) case participants from HSR2. In the end, transactional history data were received for 158 cases (88% case participant data retention rate). (Table 2)

Table 2: Number of participants included in the transactional data analysis by age and retailers

Age	HSR1				HSR2			
	Cases (N=134)		Eligible Controls (N=120)		Cases (N=130)		Eligible Controls (N=107)	
	Matched	%	Matched	%	Matched	%	Matched	%
**18-49**	9	6.7	41	34.2	10	7.6	28	26.2
**50-59**	19	14.2	31	25.8	26	20.0	25	23.4
**60-69**	43	32.1	25	20.8	43	33.1	20	18.7
**70+**	58	43.3	9	7.5	48	36.9	10	9.3
**Total**	129	96.3	106	88.3	127	97.7	83	77.6

Eligible = ID verified

To maximise the number of ID verified control participants, over 700 email reminders were sent to participants asking them to complete the ID verification step. As of 31 January 2022, 160 (37.5%) control participants provided sufficient ID verification and were eligible for purchase history requests on their behalf. At the end of the study, 137 control participant (85.6% control participant data retention rate) card details matched retailer records, allowing the research team to receive up to six years of their past transactional data (See Table 3). Only 51% of control participants had records for both retailers. The association between age distribution and ID verification among control participants was not significant (p=0.80).

For peer review only - http://bmjopen.bmj.com/site/about/guidelines.xhtml

Representativeness based on the store postcodes recorded on loyalty cards

Based on the transactions recorded on loyalty cards, we identified 697 postcodes for cases and 1127 postcodes for controls in nine UK Regions, excluding Northern Ireland. The proportion of the distribution of the purchases carried out by cases and controls in each UK region were significantly different, p<0.001 (See Table 3). More than five percentage points difference between cases and controls were recorded in transactions at East of England and Yorkshire and Humber (6.9% vs 18.5%), Midlands (4.3% vs 12.1%), South East (27.7% vs. 17.6%), and Wales (15.1% vs 3.8%) respectively. The largest proportion of purchases for both cases and controls were collected in South East.

Table 3: The number of individual transaction postcodes recorded on loyalty cards among participants in each UK region.

UK Region[t]	Transaction postcodes			
	Cases		Controls	
	N*	%	N*	%
**East of England & Yorkshire and Humber**	48	6.9	209	18.5
**London**	104	14.9	192	17.0
**North East**	24	3.4	36	3.2
**North West**	47	6.7	87	7.7
**Midlands**	30	4.3	136	12.1
**Scotland**	85	12.2	82	7.3
**South East**	193	27.7	198	17.6
**South West**	61	8.8	144	12.8
**Wales**	105	15.1	43	3.8
**Total**	697	100.0	1127	100.0

*The transactions could have been recorded at one or more store postcodes.
[t] Purchases at Northern Ireland were excluded due to small numbers (n<5)

For peer review only - http://bmjopen.bmj.com/site/about/guidelines.xhtml

However, there were no significant differences between IMD quintiles recorded for store locations where overall purchases took place between cases and controls, p=0.79 (See Table 4). Among cases, 105 transactions (20.5%) took place in stores located in the most deprived quintile (1), comparison to 188 purchases (19.0%) among controls. The lowest numbers of transactions were recorded in stores located in the least deprived quintile for both cases (n= 67, 13.1%) and controls (n=124, 12.5%).

Table 4: The number of transactions recorded on loyalty cards among participants based on the Index of Multiple Deprivation (IMD) Quintiles

Index of Multiple Deprivation Quintile	Transactions			
	Cases		Controls	
	N *	%	N*	%
**1 (Most Deprived)**	105	20.5	188	19.0
**2**	129	23.5	261	26.4
**3**	121	23.7	231	23.4
**4**	98	19.2	185	18.7
**5 (Least Deprived)**	65	13.1	124	12.5
**Total***	511	100.0	989	100.0

*No postcode-IMD conversions were recorded for the store postcodes located in Wales and Scotland.

Cost per control participant recruitment

Most controls were recruited through paid social media advertisement (n=249, 58.3%), costing on average £15 per participant compared to opportunistic methods which were not paid (n=178, See Table 5). About 31% (n=133) of control participants were recruited opportunistically during additional research that investigated attitudes towards CLOCS and willingness to take part, but their recruitment pathway was not recorded at the time

For peer review only - http://bmjopen.bmj.com/site/about/guidelines.xhtml
For peer review only

(missing data, n=131, 31%). The cost per participant for those non-targeted studies was an average of £5.

Table 5: Source from which control participants reported they heard about the study and cost of recruitment per participant from each source.

**Recruitment source**	**N**	**%**	**Cost per participant (GBP)**
From a patient	4	0.9	0.00
HSR Email	4	0.9	0.00
Twitter	2	0.5	0.00
Facebook	249	58.3	15.00
Instagram	3	0.7	0.00
Press	1	0.2	0.00
Word of Mouth	23	5.4	0.00
VOICE	8	1.9	0.00
Missing*	133	31.1	5.00
**Total**	427	100.0	

*Number of participants recruited prior to amendment to the research protocol in September 2020 that included a new item measuring the source of recruitment

Risk Factor Questionnaire Completion Rates

The majority of the items on the risk factor questionnaire were completed by both cases and controls, with the largest proportion of missing data observed for menopausal status among control participants (21.08%), followed by breastfeeding duration (18.50%), and BMI (6.79%). Both cases and controls had similar missing values for the number of full-term birth (4.40% vs. 6.09%), the number of non-full-term birth (6.59% vs 7.26%), and age at first birth (3.85% vs 4.22%). The distribution of the missing data is reported in Supplementary Table 1 (Supplementary Material 2).

DISCUSSION

For peer review only - http://bmjopen.bmj.com/site/about/guidelines.xhtml

For peer review only

The participant recruitment and data retention in this observational case-control study demonstrate that this is a feasible method and is acceptable to the public to investigate individual transactional data for health research. This is a major achievement in social sciences and epidemiological research to further understand the impact of individual behaviours on health outcomes with robust and objective datasets. Furthermore, despite the challenges of the COVID-19 pandemic, when recruitment was finalised on 31 January 2022, the study was only 73 participants short of the initial target to recruit 500 control participants. CLOCS recruited approximately 10 cases and 25 controls per month over the course of 17 active recruitment months. Most control participants were willing to take part (n=427) and provided their consent for the researchers to request their transactional loyalty card history from the high street retailers. However, the researchers were unable to process over two thirds of the control participants consent due to the lack of identity verification details. Furthermore, about 25% and 14% of cases and controls who provided information, including the name on the card and the long digit card number, did not match with the retailers' records, resulting in only 173 cases and 138 controls included in the final transactional data analyses.

The representativeness of the study population was assessed with the comparison of the population characteristics as well as the comparison of the population based on the postcode level characteristics of the store locations recorded on the loyalty cards. A key outcome is younger individuals without ovarian cancer were more willing to take part than those who were 60 years and older, despite all age groups being targeted as part of the recruitment process. This sampling bias is commonly observed in many survey studies with broad inclusion criteria for those who are eligible to take part [12] and unable to reach the

For peer review only - http://bmjopen.bmj.com/site/about/guidelines.xhtml

sample size without careful targeting. On the other hand, CLOCS case participants were representative of the age distribution of ovarian cancer patients in the UK [3] and were recruited across 12 research sites. The imbalance in age between cases and controls is a potential barrier for 1:1 case and control age-matched analyses as the majority of ovarian cancer patients were aged 60 years old and above. Therefore, it is proposed that 2:1 age matching with replacement is used adjusting for number of people in the household, and ovarian cancer risk factors that are found to be significantly associated with ovarian cancer risk among CLOCS participants. This solution to case-control matching is in line with other case-control studies with insufficient control participants to match each case [17]. It is important to note that the High Street Retailers were not significantly involved in recruitment to CLOCS. A pilot study of an email from one High Street Retailer resulted in four control participants (Table 5). The remaining control participants (n=423) were recruited directly by the researchers through various sources. However, the limitation is acknowledged that control participants who have chosen to participate might be biased due to frequent loyalty card use or more health conscious than the cancer patients which may be reflected in their shopping habits. There were also some contextual barriers as an outcome of the COVID-19 pandemic to be considered for future research. It is suggested that the way the UK government collected data to inform public health strategies may have had a negative influence on people's willingness to take part in studies like CLOCS. In July 2020, after the first lockdown restrictions were eased in the UK [18], we carried out a population-based survey in England asking individuals their willingness to share commercial data including shopping, internet searches, wearable devices, and social media data for health research [19]. A key finding of this study was that two-thirds of the population were willing to share their commercial data with academics for health research, and willingness to

For peer review only - http://bmjopen.bmj.com/site/about/guidelines.xhtml

share data reduced with the participants' ages. Also, only half of the respondents were happy to share shopping data (51.8 %) although it was more acceptable compared to internet searches (35.2%), smart phone applications (32%), wearable devices (31.8%), and social media data (30.5%) [19]. These findings are reflective of the reduction in public trust in the government and the negative perceptions towards the contact tracing app that collected personal information using mobile phone data [2021].

Under Article 15 in the General Data Protection Regulation (GDPR) in the UK, individuals have the right to access and request a copy of their data, often referred to as a subject access request [22]. There is growing literature on impersonating to take part in research for financial gains and/or trying to get access to sensitive information [23]. Transactional data is considered sensitive data and is, therefore, subject to strict data security measures by data controllers. Although ID verification is not required by all organisations, it is recommended for researchers to verify the authenticity of the participants so that data of individuals are not shared with third party organisations unlawfully. It is possible that the process of requesting ID verification details in CLOCS may not have been as clear to the participants who agreed to take part in the study resulting in dropouts. Previous studies have shown that individuals only read part of the study information and the consent form [24, 25] which may explain why 14 control participants withdrew after they were asked to provide ID verification details and a majority did not respond to our email reminders. A potential solution to reduce the number of dropouts could be to use dynamic consent processes and two-step authentication processes that are commonly used in other secure access scenarios such as online banking and shopping [26]. Dynamic consent gives the user more control over

For peer review only - http://bmjopen.bmj.com/site/about/guidelines.xhtml

For peer review only

when they want to share their data, how they want to share it, and allows researchers to amend the consent if necessary to add new variables and datasets [26].

In conclusion, this paper demonstrates the feasibility outcomes of CLOCS and discusses how to resolve the issues in the future to maximise participation and data retention rates. Specifically, it shows that although the general public is amenable to taking part and providing consent for their data to be shared with researchers, due to robust application of the data sharing principles based on GDPR and Data Protection Act 2018 by the researchers and the high street retailers, only a third of the dataset was included for the control participants. The contextual and procedural barriers as well as the public acceptability of data sharing should be considered in the design of future studies to ensure that individuals' contributions to health research with transactional data is successful.

ACKNOWLEDGEMENTS

The authors would like to acknowledge all of the participants in CLOCS, the participating retailers, and the NHS sites that recruited ovarian cancer patients to this study. We would like to acknowledge Deborah Tanner and Fiona Murphy who have acted as ovarian cancer patient representatives since the inception of the CLOCS project. This study was funded by Cancer Research UK Early Detection and Diagnosis project grant (C38463/A26726) with generous support from the Peter Sowerby Foundation and additional support from the Cancer Research UK Imperial Centre, the National Institute for Health Research Imperial Biomedical Research Centre and the Ovarian Cancer Action Research Centre. The authors have no conflicts of interest to declare.

CONFLICT OF INTEREST STATEMENT

For peer review only - http://bmjopen.bmj.com/site/about/guidelines.xhtml

The authors declare no conflicts of interest for this study.

AUTHOR CONTRIBUTIONS

JMF, YH, HRB conceived the study. HRB and YH conducted all statistical analyses. HRB, JMF and YH reviewed the results. EJ provided expertise in information governance and data management. HRB wrote the first draft of the manuscript. YH, JMF, SS and MCH commented and reviewed the final draft of the manuscript. All authors agree on the final version of the manuscript.

DATA SHARING STATEMENT

Access to all CLOCS purchase history data collected from the retailers is restricted due to the data sharing agreements in place and the sensitive nature of the data. We, however, welcome other researchers to contact us for collaborations. The PI and research team will review each proposal and, if approved, the analysis will be conducted by a member of the CLOCS team.

ETHICAL APPROVAL

CLOCS was reviewed and approved by the North West-Greater Manchester South Research Ethics Committee (19/NW/0427). Explicit consent for the research team to request loyalty card data from participating retailers and to take part in the study was given from all participants.

TRANSPARENCY STATEMENT

All authors confirm that the manuscript is an honest, accurate, and transparent account of the study, and no important aspects of the study have been omitted.

For peer review only - http://bmjopen.bmj.com/site/about/guidelines.xhtml

FUNDING

The Cancer Loyalty Card Study (CLOCS) is funded by Cancer Research UK (CRUK) Early Detection Project Grant (C38463/A26726) with generous support from the Peter Sowerby Foundation. YH and HRB are funded by CRUK.

For peer review only

REFERENCES

1. Smittenaar, C., Petersen, K., Stewart, K. et al. Cancer incidence and mortality projections in the UK until 2035. Br J Cancer 2016. 115, 1147–1155. https://doi.org/10.1038/bjc.2016.304
2. Funston G, Hamilton W, Abel G, Crosbie EJ, Rous B, Walter FM. The diagnostic performance of CA125 for the detection of ovarian and non-ovarian cancer in primary care: A population-based cohort study. PLoS Med. 2020 Oct 28;17(10):e1003295. doi: 10.1371/journal.pmed.1003295
3. Cancer Research UK. 2022. Ovarian cancer statistics. [online] Available at: <https://www.cancerresearchuk.org/health-professional/cancer-statistics/statistics-by-cancer-type/ovarian-cancer> [Accessed 10 May 2022].

4. Barr CE, Funston G, Jeevan D, Sundar S, Mounce LTA, Crosbie EJ. The Performance of HE4 Alone and in Combination with CA125 for the Detection of Ovarian Cancer in an Enriched Primary Care Population. Cancers (Basel). 2022 Apr 24;14(9):2124. doi: 10.3390/cancers14092124.

5. Davenport C, Rai N, Sharma P, Deeks JJ, Berhane S, Mallett S, Saha P, Champaneria R, Bayliss SE, Snell KI, Sundar S. Menopausal status, ultrasound and biomarker tests in combination for the diagnosis of ovarian cancer in symptomatic women. Cochrane Database Syst Rev. 2022 Jul 26;7(7):CD011964. doi: 10.1002/14651858.CD011964.pub2.

6. Menon U, Gentry-Maharaj A, Burnell M, Singh N, Ryan A, Karpinskyj C, Carlino G, Taylor J, Massingham SK, Raikou M, Kalsi JK, Woolas R, Manchanda R, Arora R, Casey L, Dawnay A, Dobbs S, Leeson S, Mould T, Seif MW, Sharma A, Williamson K, Liu Y, Fallowfield L, McGuire AJ, Campbell S, Skates SJ, Jacobs IJ, Parmar M. Ovarian cancer population screening and mortality after long-term follow-up in the UK Collaborative Trial of Ovarian Cancer Screening (UKCTOCS): a randomised controlled trial. Lancet. 2021 Jun 5;397(10290):2182-2193. doi: 10.1016/S0140-6736(21)00731-5.

7. Sundar S, Rick C, Dowling F on behalf of the ROCkeTS study group, et al. Refining Ovarian Cancer Test accuracy Scores (ROCkeTS): protocol for a prospective longitudinal test accuracy study to validate new risk scores in women with symptoms of suspected ovarian cancer. BMJ Open 2016;6:e010333. doi: 10.1136/bmjopen-2015-010333

8. Radu CA, Matos de Melo Fernandes N, Khalfe S, Stordal B. Awareness of ovarian cancer symptoms and risk factors in a young ethnically diverse British population. Cancer Med. 2023 Feb 7. doi: 10.1002/cam4.5670.

For peer review only - http://bmjopen.bmj.com/site/about/guidelines.xhtml

9. Brewer HR, Hirst Y, Sundar S, et al. Cancer Loyalty Card Study (CLOCS): protocol for an observational case-control study focusing on the patient interval in ovarian cancer diagnosis. BMJ Open Published Online First: 2020. doi:10.1136/bmjopen-2020-037459

10. Brewer HR, Hirst Y, Chadeau-Hyam M, Johnson E, Sundar S, Flanagan JM. Association Between Purchase of Over-the-Counter Medications and Ovarian Cancer Diagnosis in the Cancer Loyalty Card Study (CLOCS): Observational Case-Control Study. JMIR Public Health Surveill. 2023 Jan 26;9:e41762. doi: 10.2196/41762.

11. Flanagan J, Skrobanski H, Shi X, et al. Self-Care Behaviors of Ovarian Cancer Patients Before Their Diagnosis: Proof-of-Concept Study. JMIR Cancer 2019;5:e10447. doi:10.2196/CANCER.10447

12. Grimes DA, Schulz KF. Compared to what? Finding controls for case-control studies. The Lancet. 2005 Apr 16;365(9468):1429-33.

13. Bernstein L. Control recruitment in population-based case-control studies. Epidemiology. 2006 May 1;17(3):255-7.

14. VOICE. 2022. Imperial College London - VOICE. [online] Available at: <https://www.voice-global.org/about/imperial-college-london/.> [Accessed 10 May 2022].

15. Imd-by-postcode.opendatacommunities.org. 2022. English indices of deprivation 2019: Postcode Lookup. [online] Available at: <https://imd-by-postcode.opendatacommunities.org/imd/2019> [Accessed 10 May 2022].

16. Bath.ac.uk. 2022. [online] Available at: <https://www.bath.ac.uk/publications/undergraduate-bursaries-and-scholarships-

For peer review only - http://bmjopen.bmj.com/site/about/guidelines.xhtml

terms-and-conditions-2020-21/attachments/Indices-of-Multiple-Deprivation-Lookup-Guide.pdf> [Accessed 10 May 2022].

17. Pearce N. Analysis of matched case-control studies. BMJ. 2016 Feb 25;352.

18. Instituteforgovernment.org.uk. 2022. [online] Available at: <https://www.instituteforgovernment.org.uk/charts/uk-government-coronavirus-lockdowns> [Accessed 10 May 2022].

19. Hirst Y, Stoffel ST, Brewer HR, Timotijevic L, Raats M, Flanagan JM. Understanding Public Attitudes and Willingness to Share Commercial Data for Health Research: A Survey Study in the United Kingdom. JMIR Public Health Surveill., in press

20. How has COVID-19 affected trust in scientists? [Internet]. IPSOS MORI; 2022 [cited 10 May 2022]. Available from: https://www.ukri.org/wp-content/uploads/2020/09/UKRI-271020-COVID-19-Trust-Tracker.pdf

21. Samuel G, Roberts SL, Fiske A, Lucivero F, McLennan S, Phillips A, Hayes S, Johnson SB. COVID-19 contact tracing apps: UK public perceptions. Critical Public Health. 2022 Jan 1;32(1):31-43.

22. GDPRhub. 2022. Article 15 GDPR. [online] Available at: <https://gdprhub.eu/Article_15_GDPR> [Accessed 10 May 2022].

23. Heckathorn DD, Broadhead RS, Sergeyev B. A methodology for reducing respondent duplication and impersonation in samples of hidden populations. Journal of Drug Issues. 2001 Apr;31(2):543-64.

24. Flory J, Emanuel E. Interventions to improve research participants' understanding in informed consent for research: a systematic review. Jama. 2004 Oct 6;292(13):1593-601.

For peer review only - http://bmjopen.bmj.com/site/about/guidelines.xhtml

25. D'Abramo F, Schildmann J, Vollmann J. Research participants' perceptions and views on consent for biobank research: a review of empirical data and ethical analysis. BMC Med Ethics. 2015;16:60.

26. Budin-Ljøsne I, Teare HJ, Kaye J, Beck S, Bentzen HB, Caenazzo L, Collett C, D'Abramo F, Felzmann H, Finlay T, Javaid MK. Dynamic consent: a potential solution to some of the challenges of modern biomedical research. BMC medical ethics. 2017 Dec;18(1):1-0.

For peer review only

Figure 1. CLOCS recruitment timeline for cases and controls.

Figure 2. Timeline of when CLOCS case participants were diagnosed with ovarian cancer by quarter (Q) and year.

Figure 3. CLOCS Consort Diagram detailing participant recruitment

For peer review only - http://bmjopen.bmj.com/site/about/guidelines.xhtml

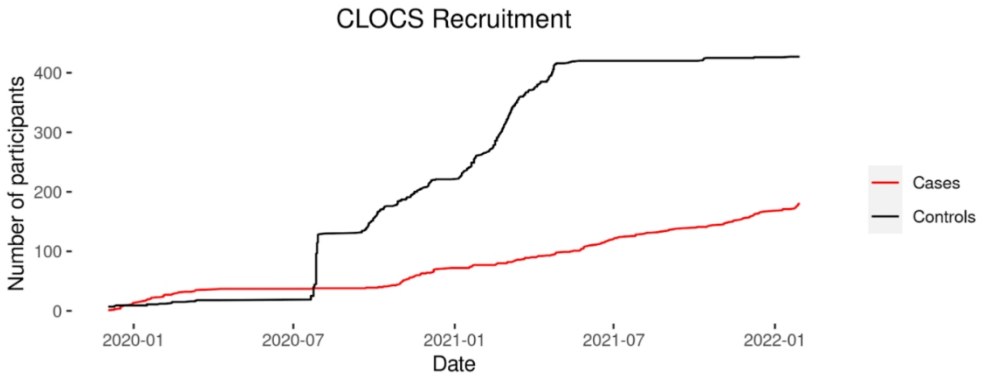

Figure 1

159x61mm (220 x 220 DPI)

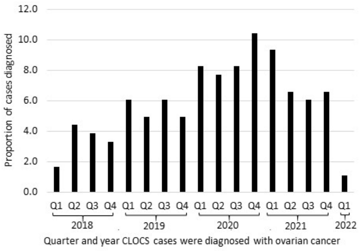

Figure 2

338x190mm (96 x 96 DPI)

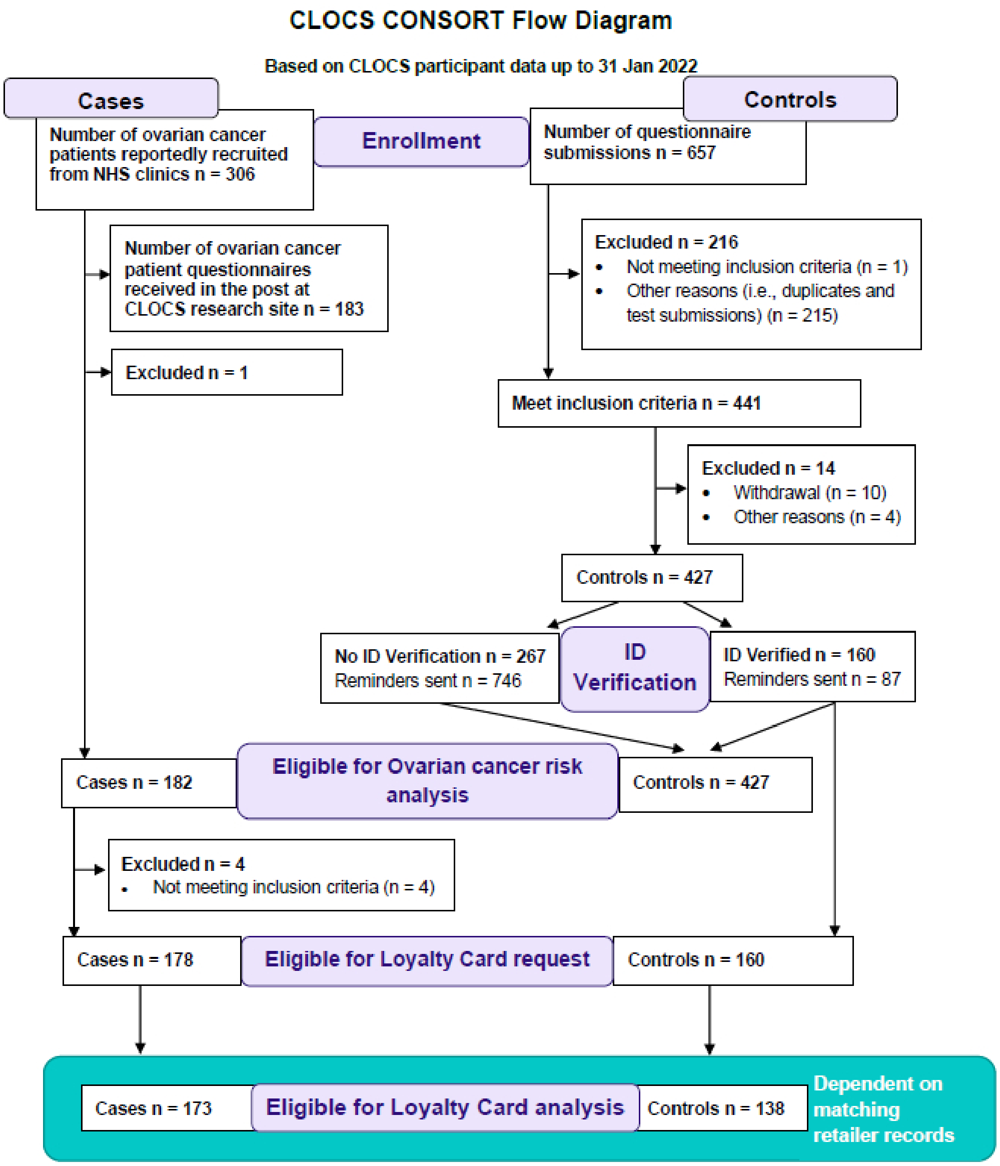

Figure 3

174x203mm (96 x 96 DPI)

Participant number:
Attach barcode here

CRUK Cancer Loyalty Card Study (CLOCS)
Participant Questionnaire

Thank you for agreeing to complete this questionnaire.

- It should take no longer than 10-15 minutes to complete. You may, however, take as long as you wish.
- Please try to answer all of the questions, but if you do not wish to answer a question, you do not have to and you can skip to the next one.
- Your answers will be sent directly to the Imperial College research team and will be treated in strict confidence.
- There are four brief sections in this questionnaire:
    1. About you
    2. Your reproductive history
    3. Your medical history
    4. Your risk perception and health status
    5. About your loyalty card use
- Please tick the appropriate box where this option is given, or write in the box given.

e.g. | ✓ | or | 1 | 3 |

For peer review only - http://bmjopen.bmj.com/site/about/guidelines.xhtml

CLOCS
Cancer Loyalty Card Study

Participant number:
Attach barcode here

Today's Date: d d / m m / y y y y

1. About you

Date of birth: d d / m m / y y y y

Which of these best describes your ethnic group?

White
British ☐ Irish ☐ Any other white background ☐

Black or Black British
Caribbean ☐ African ☐ Any other Black background ☐

Asian or Asian British
Indian ☐ Pakistani ☐ Bangladeshi ☐ Any other Asian background ☐

Mixed
White and Black Caribbean ☐ White and Black African ☐ White and Asian ☐
Any other Mixed background ☐

Other Ethnic Groups
Chinese ☐ Any other ethnic group ☐ Prefer not to say ☐

What is your marital status?

Single / never married ☐ Married / living with partner ☐ Divorced / separated ☐
Widowed ☐ Prefer not to say ☐

What is your current height? ☐☐☐ cm or ☐ ft ☐☐ in

What is your current weight? ☐☐☐ kg or ☐☐ st ☐☐ lb

Including yourself, how many people live in your household? ☐ people

For peer review only

CLOCS Cancer Loyalty Card Study

Participant number:
Attach barcode here

2. Your reproductive history

How old were you when you had your first period? [ ][ ] years **or** I have never had a period [ ]

Do you currently have regular periods?

Yes [ ] No [ ] **If no**, what was your age at menopause? [ ][ ] years **or** irregular [ ]

Not applicable, I have never had a period [ ]

Have you ever been pregnant before? Yes [ ] No [ ]

If yes:

How many pregnancies have you had that lasted less than 37 weeks? [ ][ ]

How many pregnancies have you had that lasted 37 weeks or more? [ ][ ]

How old were you when you first gave birth? [ ][ ] years

How old were you when you last gave birth? [ ][ ] years

Have you ever breastfed your children? Yes [ ] No [ ] Not applicable [ ]

If yes:

For how many months (total for all children)? [ ][ ] months

Have you had a hysterectomy (i.e. removal of your womb)? Yes [ ] No [ ]

If yes:

At what age did you have a hysterectomy? [ ][ ] years

Have you had tubal ligation (i.e. sterilization, tubes tied)? Yes [ ] No [ ]

If yes:

At what age did you have tubal ligation? [ ][ ] years

For peer review only - http://bmjopen.bmj.com/site/about/guidelines.xhtml

CLOCS
Cancer Loyalty Card Study

Participant number:
Attach barcode here

3. Your medical history

In the past 12 months, have you experienced any of these symptoms for an extended amount of time? (tick all that apply)

Feeling constantly bloated	☐	Loss of appetite	☐	Pain during sex	☐	Feeling full quickly	☐
Swollen Tummy	☐	Urge to pee more often or urgently than usual	☐	Constipation	☐	Feeling tired all the time	☐
Discomfort in lower tummy or pelvic area	☐	Persistent nausea	☐	Back pain	☐	Persistent indigestion	☐
Irregular periods	☐	Vaginal bleeding after menopause	☐	Unintentional weight loss	☐	None of these	☐

Did you use any over-the-counter (non-prescription) medication to manage these symptoms before seeing your GP?

Yes ☐ No ☐ Waited for the symptom to resolve on its own ☐ Not applicable ☐

How many times did you visit your GP in the past year? ☐☐ times

Have you ever been diagnosed with endometriosis? Yes ☐ No ☐ I don't know ☐

If yes:

At what age were you diagnosed with endometriosis? ☐☐ years

Have you ever taken aspirin regularly? Yes ☐ No ☐

If yes:

How often? Daily ☐ Weekly ☐ Less often ☐

For how long have you taken aspirin regularly? ☐☐ years and/or ☐☐ months

What dose of aspirin did you take?

Low dose (usually about 75mg/day) ☐ High dose (usually about 300mg/day) ☐ I don't know ☐

Have you ever used oral contraceptive pills (also called 'the pill' or 'birth control pill')? Yes ☐ No ☐

If yes:

For how long? ☐☐ years and/or ☐☐ months

For peer review only

CLOCS Cancer Loyalty Card Study

Participant number:
Attach barcode here

Your medical history continued

Have you ever used hormone replacement therapy? Yes ☐ No ☐

If yes:

For how long? ☐☐ years and/or ☐☐ months

Have you ever been diagnosed with cancer? Yes ☐ No ☐

If yes, what type? (tick all that apply):

Breast	☐	Melanoma	☐	Head and Neck	☐	Pancreas	☐	Uterus	☐
Bowel	☐	Non-Hodgkin Lymphoma	☐	Brain	☐	Leukemia	☐	Endometrial	☐
Lung	☐	Kidney	☐	Bladder	☐	Oesophagus	☐	Stomach	☐
Liver	☐	Myeloma	☐	Thyroid	☐	Ovarian	☐	Skin (non-melanoma)	☐

Other, please specify: ____

Please list any cancer(s) ticked above and the year in which you were diagnosed:

Type:________________________________ Year diagnosed: ☐☐☐☐

Type:________________________________ Year diagnosed: ☐☐☐☐

Do you have a mother, sister, or daughter with a history of <u>ovarian</u> cancer? Yes ☐ No ☐

If yes, in which relative(s)? Mother ☐ Sister ☐ Daughter ☐

Another sister or daughter, please specify relationship: ______________________ ☐ ______________________ ☐

Do you have a mother, sister, or daughter with a history of <u>breast</u> cancer? Yes ☐ No ☐

If yes, in which relative(s)? Mother ☐ Sister ☐ Daughter ☐

Another sister or daughter, please specify relationship: ______________________ ☐ ______________________ ☐

For peer review only - http://bmjopen.bmj.com/site/about/guidelines.xhtml

CLOCS
Cancer Loyalty Card Study

Participant number:
Attach barcode here

Your medical history continued

Have you ever regularly smoked cigarettes? Yes ☐ No ☐

If yes:

For how many years? ☐☐ years

Do you still smoke regularly? Yes ☐ No ☐

Have you ever regularly used vaping products? Yes ☐ No ☐

If yes:

For how many years? ☐☐ years

Do you still vape regularly? Yes ☐ No ☐

Due to the unprecedented Covid-19 (coronavirus) outbreak and lockdown started in March 2020 in the UK, our analysis of the past purchase information during the lockdown in the UK will be more complex than it would have been. We would like to be able to make sure we can control for the impact of Covid-19 on our analyses.

Have you had Covid-19 (coronavirus)?

Yes, diagnosed and recovered ☐ Yes, diagnosed and still ill ☐ Not formally diagnosed, but suspected ☐ No / Not that I know of ☐

If yes, please state the approximate date you presented with symptoms below:

d d / m m / y y y y

4. Your risk perception and health status

Compared to most other women your age, how likely do you think it is that you will get ovarian cancer at some time in your life?

Much lower than others ☐ Lower than others ☐ The same as others ☐ Higher than others ☐ Much higher than others ☐

How confident are you that you would notice an ovarian cancer symptom?

Not at all confident ☐ Not very confident ☐ Fairly confident ☐ Very confident ☐

Would you say your health is:

Excellent ☐ Very good ☐ Good ☐ Fair ☐ Poor ☐

Imperial College London

CANCER RESEARCH UK

For peer review only - http://bmjopen.bmj.com/site/about/guidelines.xhtml

CLOCS
Cancer Loyalty Card Study

Participant number:
Attach barcode here

5. About your loyalty card use

At which food and pharmacy stores do you shop most regularly? (tick all that apply)

Tesco ☐ Sainsbury's ☐ Waitrose ☐ ASDA ☐ Boots ☐ Superdrug ☐
Co-op ☐ Morrison's ☐ Other, please specify: ______________________

At which food and pharmacy stores do you own a loyalty card? (tick all that apply)

Tesco ☐ Sainsbury's ☐ Waitrose ☐ ASDA ☐ Boots ☐ Superdrug ☐
Co-op ☐ Morrison's ☐ Other, please specify: ______________________

How often do you use loyalty cards when you go shopping?

Not at all ☐ Not very often ☐ Sometimes ☐ Often ☐ All the time ☐

How did you hear about this study?

From a patient ☐ Twitter ☐ Facebook ☐ Instagram ☐ Press ☐ Word of mouth ☐

Thank you for completing this form.

- Please return the survey in the freepost envelope (no stamp required).

For peer review only

Cancer Loyalty Card Study (CLOCS): feasibility outcomes for an observational case-control study focusing on the patient interval in ovarian cancer

Hannah R. Brewer[1], Marc Chadeau-Hyam[2], Eric Johnson[2], Sudha Sundar[3], James M. Flanagan[1*] and Yasemin Hirst[4]

SUPPLEMENTARY MATERIAL

Supplementary Table 1: Ovarian Cancer Risk questionnaire item distribution and missing data

Risk factor questionnaire items	Cases		Controls	
	N	%	N	%
**Age (years)**	182	100.00	422	98.83
Missing	*0*	*0.00*	*5*	1.17
**Age at menarche (years)**				
08-11	38	20.88	95	22.25
12	43	23.63	89	20.84
13	39	21.43	101	23.65
14-19	57	31.32	138	32.32
Missing	*5*	2.75	*4*	0.94
**OC use**				
Never	43	23.63	50	11.71
Ever	138	75.82	377	88.29
Missing	*1*	0.55	*0*	0.00
**Duration of OC use (years)**				
0	52	28.57	56	13.11
1-5	59	32.42	140	32.79
>5	71	39.01	231	54.10
Missing	*0*	0.00	*0*	0.00
**Parity**				
Never	42	23.10	109	25.53
Ever	139	76.40	316	74.00
Missing	*1*	*0.50*	*2*	0.47
**No. full-term births**				

For peer review only - http://bmjopen.bmj.com/site/about/guidelines.xhtml

0	55	30.22	138	32.32
1	29	15.93	70	16.39
2	59	32.42	129	30.21
≥3	31	17.03	64	14.99
Missing	*8*	4.40	*26*	6.09
**No. non full-term births**				
0	125	68.68	244	57.14
1	29	15.93	97	22.72
2	10	5.49	29	6.79
>=3	6	3.30	26	6.09
Missing	*12*	6.59	*31*	7.26
**Age last birth**				
Never	49	26.92	128	29.98
<=25	25	13.74	45	10.54
26-30	44	24.18	83	19.44
>=31	57	31.32	153	35.83
Missing	*7*	3.85	*18*	4.22
**Breastfeeding**				
Never	94	51.65	202	47.31
Ever	87	47.80	220	51.52
Missing	*1*	0.55	*5*	1.17
**Breastfeeding duration (months)**				
Never	96	52.75	144	33.72
01-6	43	23.63	92	21.55
07-12	15	8.24	33	7.73
>12	27	14.84	79	18.50
Missing	*0*	0.00	*79*	18.50
**Tubal ligation**				
Never	154	84.62	376	88.06
Ever	22	12.09	51	11.94
Missing	*6*	3.30	*0*	0.00
**Endometriosis**				
Not diagnosed	170	93.41	393	92.04
Diagnosed	12	6.59	34	7.96
Missing	*0*	0.00	*0*	0.00
**Family history of breast cancer (first-degree relative)**				
None	150	82.42	366	85.71
Yes	31	17.03	60	14.05
Missing	*1*	0.55	*1*	0.23
**Family history of ovarian cancer (first-degree relative)**				
None	166	91.21	379	88.76
Yes	16	8.79	46	10.77

For peer review only - http://bmjopen.bmj.com/site/about/guidelines.xhtml

Missing	*0*	0.00	*2*	0.47
**BMI**				
Underweight (BMI < 18.5)	5	2.75	4	0.94
Normal (18.5 – 25)	75	41.21	138	32.32
Overweight (25-30)	50	27.47	131	30.68
Obese (>30)	44	24.18	125	29.27
Missing	*8*	4.40	*29*	6.79
**Aspirin use**				
Irregular or no use	159	87.36	390	91.33
Regular user of low-dose aspirin	18	9.89	28	6.56
Regular user of high-dose aspirin	3	1.65	1	0.23
Missing	*2*	1.10	*8*	1.87
**Menopausal status**				
Premenopausal	8	4.40	106	24.82
Postmenopausal	163	89.56	231	54.10
Missing	*11*	6.04	*90*	21.08
**Hysterectomy**				
Never	45	24.73	399	93.44
Ever	133	73.08	26	6.09
Missing	*4*	2.20	*2*	0.47
**HRT use**				
Never	123	67.58	343	80.33
Ever	58	31.87	84	19.67
Missing	*1*	0.55	*0*	0.00
**Duration of HRT use (years)**				
0	124	68.13	344	80.56
1-5	31	17.03	58	13.58
>5	27	14.84	21	4.92
Missing	*0*	0.00	*3*	0.70
**Smoking**				
Never	102	56.04	261	61.12
Ever	80	43.96	165	38.64
Missing	*0*	0.00	*1*	0.23
**Duration of Smoking (years)**				
0	102	56.04	261	61.12
1-10	27	14.84	55	12.88
>10-20	21	11.54	47	11.01
>20	32	17.58	60	14.05
Missing	*0*	0.00	*4*	0.94
**Vaping**				
Never	173	95.05	394	92.27
Ever	9	4.95	32	7.49
Missing	*0*	0.00	*1*	0.23

For peer review only - http://bmjopen.bmj.com/site/about/guidelines.xhtml

Duration of Vaping (years)				
0	173	95.05	394	92.27
1-5	7	3.85	27	6.32
>5	2	1.10	4	0.94
Missing	*0*	0.00	*2*	0.47

For peer review only

For peer review only - http://bmjopen.bmj.com/site/about/guidelines.xhtml

STROBE Statement—Checklist of items that should be included in reports of ***case-control studies***

	Item No	Recommendation	Page No
**Title and abstract**	1	(*a*) Indicate the study's design with a commonly used term in the title or the abstract	2
		(*b*) Provide in the abstract an informative and balanced summary of what was done and what was found	3
**Introduction**			
Background/rationale	2	Explain the scientific background and rationale for the investigation being reported	4
Objectives	3	State specific objectives, including any prespecified hypotheses	4
**Methods**			
Study design	4	Present key elements of study design early in the paper	4
Setting	5	Describe the setting, locations, and relevant dates, including periods of recruitment, exposure, follow-up, and data collection	4-5
Participants	6	(*a*) Give the eligibility criteria, and the sources and methods of case ascertainment and control selection. Give the rationale for the choice of cases and controls	5
		(*b*) For matched studies, give matching criteria and the number of controls per case	7
Variables	7	Clearly define all outcomes, exposures, predictors, potential confounders, and effect modifiers. Give diagnostic criteria, if applicable	6-7
Data sources/ measurement	8*	For each variable of interest, give sources of data and details of methods of assessment (measurement). Describe comparability of assessment methods if there is more than one group	7
Bias	9	Describe any efforts to address potential sources of bias	7
Study size	10	Explain how the study size was arrived at	Published (Brewer et al BMJ Open 2020)
Quantitative variables	11	Explain how quantitative variables were handled in the analyses. If applicable, describe which groupings were chosen and why	7
Statistical methods	12	(*a*) Describe all statistical methods, including those used to control for confounding	6-7
		(*b*) Describe any methods used to examine subgroups and interactions	7
		(*c*) Explain how missing data were addressed	6
		(*d*) If applicable, explain how matching of cases and controls was addressed	7
		(*e*) Describe any sensitivity analyses	7
**Results**			
Participants	13*	(a) Report numbers of individuals at each stage of study—eg numbers potentially eligible, examined for eligibility, confirmed eligible, included in the study, completing follow-up, and analysed	Figure 1 and page 8
		(b) Give reasons for non-participation at each stage	8
		(c) Consider use of a flow diagram	Figure 1
Descriptive data	14*	(a) Give characteristics of study participants (eg demographic, clinical,	Table 1, page

For peer review only

		social) and information on exposures and potential confounders	20
		(b) Indicate number of participants with missing data for each variable of interest	Supplementary Tables
Outcome data	15*	Report numbers in each exposure category, or summary measures of exposure	Supplementary Tables

For peer review only

Main results	16	(*a*) Give unadjusted estimates and, if applicable, confounder-adjusted estimates and their precision (eg, 95% confidence interval). Make clear which confounders were adjusted for and why they were included	9
		(*b*) Report category boundaries when continuous variables were categorized	NA
		(*c*) If relevant, consider translating estimates of relative risk into absolute risk for a meaningful time period	NA
Other analyses	17	Report other analyses done—eg analyses of subgroups and interactions, and sensitivity analyses	9-10
**Discussion**			
Key results	18	Summarise key results with reference to study objectives	10
Limitations	19	Discuss limitations of the study, taking into account sources of potential bias or imprecision. Discuss both direction and magnitude of any potential bias	12
Interpretation	20	Give a cautious overall interpretation of results considering objectives, limitations, multiplicity of analyses, results from similar studies, and other relevant evidence	13
Generalisability	21	Discuss the generalisability (external validity) of the study results	13
**Other information**			
Funding	22	Give the source of funding and the role of the funders for the present study and, if applicable, for the original study on which the present article is based	16

*Give information separately for cases and controls.

Note: An Explanation and Elaboration article discusses each checklist item and gives methodological background and published examples of transparent reporting. The STROBE checklist is best used in conjunction with this article (freely available on the Web sites of PLoS Medicine at http://www.plosmedicine.org/, Annals of Internal Medicine at http://www.annals.org/, and Epidemiology at http://www.epidem.com/). Information on the STROBE Initiative is available at http://www.strobe-statement.org.

For peer review only - http://bmjopen.bmj.com/site/about/guidelines.xhtml
